# Synthesis of diverse spiro-imidazo pyridine-indene derivatives via acid-promoted annulation reaction of bindone and heterocyclic ketene aminals

**DOI:** 10.1038/s41598-022-16959-w

**Published:** 2022-07-22

**Authors:** Shima Nasri, Mohammad Bayat, Fatemeh Rostami Miankooshki

**Affiliations:** Department of Chemistry, Faculty of Science, Imam Khomeini International University, Qazvin, Iran

**Keywords:** Organic chemistry, Chemical synthesis

## Abstract

A new multi-component reaction for the synthesis of novel and diverse spiro-imidazo pyridine-indene derivatives named spiro[imidazo[1,2-*a*]indeno[2,1-*e*]pyridine-5,1′-indene and indenylidene-1*H*-spiro[imidazo[1,2-*a*]pyridine-7,1′-indene was successfully developed by the reaction between heterocyclic ketene aminals (generated from 1,1-bis(methylthio)-2-nitro ethylene and diamine) and [1,2′-biindenylidene]-1′,3,3′-trione (bindone) (in situ generated from self-condensation of 1,3-indandion) by using malononitrile as a promoter or as one of the precursors respectively in the presence of *p*-TSA as the acid catalyst in EtOH as reaction medium under reflux conditions. Depending on whether the reaction is single-step or two-step, malononitrile can act as a promoter or reactant. The convenient one-pot operation, straightforward isolation without using additional purification methods, and the use of a variety of diamines and cysteamine hydrochloride causing a variety of structural products are attractive aspects of the present approach. The synthesized bindone and final product contains active methylene and this active site can be involved in further reactions to synthesize more complex heterocycles.

## Introduction

**Figure 1:**
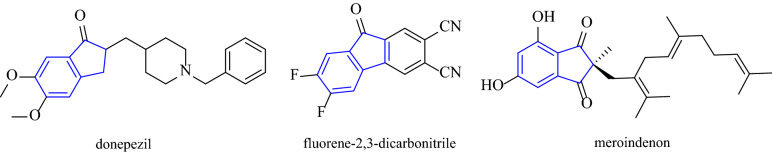
Selective indenone-fused heterocycles with medicinal activity.

Among the carbocyclic and heterocyclic products, indenone-fused compounds are important structures with a great variety of pharmaceutical activities. Indanones have now been introduced as important scaffolds of several natural compounds, important pharmaceutical products, biological materials, agrochemicals, and functionalized substances^1,2^. Among the pharmacological significant indanones, the most important medicine possibly is donepezil, an acetylcholinesterase inhibitor for the therapy of Alzheimer's disease^3^ and it is observed in various other therapeutic candidates with different clinical activities^4,5^. For example, fluorene compound is an efficient therapy of drug-resistant nonsmall-cell lung cancer^6^, meroindenon was isolated from a marine-derived bacterium belonging to the genus Streptomyces^7,8^ (Fig. 1).

Due to the presence of three contiguous electrophilic and nucleophilic reactive centers in the structure of 1,3-indanedione as a cyclic 1,3-dicarbonyl scaffold and for its features including easy to handle, low cost, participating in environmentally friendly methods that generally cause the corresponding products in excellent yields^9^, 1,3-indanedione is a beneficial precursor for the synthesis of various indanone-containing polycyclic products^9,10^. It is noteworthy that the base- or acid-catalyzed self-condensation of 1,3-indanedione creates the active dimer of 1,3-indanedione (bindone) and the cyclotrimer of 1,3-indanedione (truxenone) and also further various oligomers depending on the reaction conditions^11^. The bindone compound is a potential dipolar donor–acceptor dye in solar cells^12^ and is a promising electron-accepting group for push–pull conjugated systems with photochemically switchable second harmonics generation^13^.

However, the self-condensation of ketones is restricted to a few reports and specific reagents and tedious reaction conditions are necessary for their practical condensation, particularly for non-activated cyclic and ketones with high molecular weight^14,15^. For example, the needed strong bases including lithium diisopropylamide and sodium hydride^16^, which these strong bases are incompatible with protic solvents, accordingly tetrahydrofuran is often applied as a medium for these reactions, which is not environmentally friendly^17^. In addition, in these reaction conditions, moisture must be completely removed from the reaction medium under an inert atmosphere. Strong acids, like hydrochloric acid and polyphosphoric acid are also used to promote the self-condensation of ketones, but usually, two to three equivalents of acid are needed to advance the reaction^15^. Other introduced techniques for the self-aldol condensation of aromatic and aliphatic ketones need organometallic^18^ or titanium alkoxides^19^, while cyclic ketone self-condensation has been reported applying a W(CO)_6_/CCl_4_/UV condition^20^. The cationic rhodium complex [Cp*Rh(ƞ^6^-C_6_H_6_)](BF_4_)_2_ is also introduced to promote the self-condensation of ketones^21^. While some of these procedures create considerable amounts of hazardous metals and harmful solvents. The absence of a general approach for the selective self-condensation of non-activated ketones under mild conditions restricts its application in organic synthesis.

**Figure 2:**
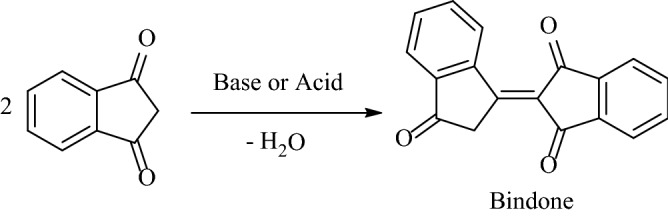
Condensation of 1,3-indanedione in basic or acidic conditions.

**Figure 3:**
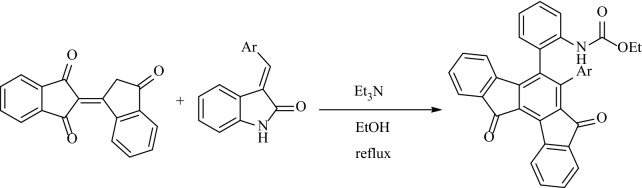
Synthetic approach for the formation of indeno[1,2-*a*]fluorene-7,12-diones using bindone.

**Figure 4:**
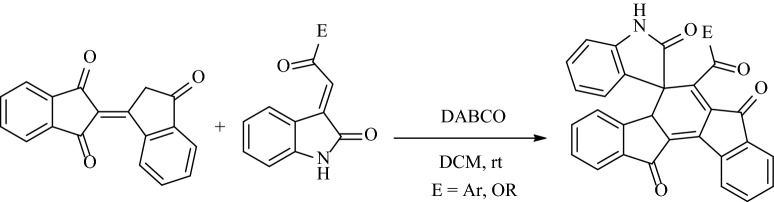
Syntheses of polycyclic spiro compounds using bindone.

The self-condensation of 1,3-indandione was considered in both basic and acidic conditions. The carbon at position C-2 is alpha to both carbonyls and so can involve in a reaction as a nucleophile. It undergoes self-aldol condensation leading to the generation of bindone^11^ (Fig. 2). Because of the synthetic importance of bindone as a valuable procedure, numerous procedures have been developed for the use of bindone for the synthesis of indanone-containing polycyclic compounds as follows: in 2019, Yan et al. have developed an efficient synthetic approach for new indeno[1,2-*a*]fluorene products by the base promoted domino reaction of 1,3-indanedione with 3-arylideneindolin-2-ones in various solvents (Fig. 3)^22^. In 2021, Yan and coworkers have reported a selective synthesis of spiro[benzo[5,6]pentaleno[1,6*a*-*b*]naphthalene-7,3*'*-indoline] derivatives and complex dispiro[indoline-3,6*'*-[4*b*,6*a*]-ethanoindeno[1,2-*a*]fluorene-14*'*,3*''*-indolines] via a DABCO-promoted annulation reaction of bindone and 3-methyleneoxindoles in acetonitrile at various temperatures for the in optimized yields (Fig. 4)^23^.

These reactions obviously demonstrated that 1,3-indanedione and its dimer are involved in cycloaddition reactions with diverse reactivity and different complex polycyclic products can be conveniently synthesized by applying available precursors. On the other, increasing environmental concern around energy efficiency and waste management provides an opportunity to develop even more powerful and greener strategies for valuable organic reactions. In this regard, and in continuation of our recent reports for the synthesis of versatile spiro and fused cyclic compounds by multi-components reactions of 1,3-indanedione^24–26^, we were looking for a general strategy for the self-condensation of indandione in EtOH using *p*-TSA as an acidic and mild catalyst. First, the reaction did not proceed in the presence of the *p*-TSA, and in the next attempt to order the condensation of malononitrile with 1,3-indandione, we added the malononitrile to the reaction medium, which succeeded in synthesizing spiro[imidazo[1,2-*a*]indeno[2,1-*e*]pyridine-5,1*'*-indene and we accidentally realized the role of malononitrile as a promoter is essential for the production of bindone. In the following, the acid-promoted annulation reaction of bindone with heterocyclic ketene aminals (HKAs) in a one-pot reaction process under different reaction conditions. HKA has been used as a readily accessible and versatile synthon for the efficient synthesis of highly functionalized heterocycles^27,28^. During the past decades, various procedures have been reported for the synthesis of substituted heterocyclic products based on using HKAs as precursors as follows: in 2014, Alizadeh et al. have reported a catalyst-free, one-pot, four-component reaction between aromatic aldehydes, cyclic 1,3-diones, diamines, and nitro ketene dithioacetal in stoichiometric melt conditions for the synthesis of octahydro-imidazo[1,2-*a*]quinolin-6-ones from^29^. In 2013, Alizadeh and co-workers have developed an efficient, one-pot synthetic procedure for the formation of polyfunctionalized 1,4-dihydropyridine-fused-1,3-diazaheterocycles using 1,1-bis(methylthio)-2-nitroethylene, 1,n-diamine, arylaldehyde, and malononitrile^30^. In 2016, Mohammadi and co-workers have synthesized 1*H*-imidazol[1,2-*a*]indeno[2,1-*e*]pyridine-6(5*H*)-one derivatives via a one-pot four-component condensation of aldehydes, 1,3-indandione, diamine, and nitro ketene dithioacetal using KAl(SO_4_)_2_.12H_2_O (alum) in good to excellent yields^31^. In 2017, Mohammadi et al. have described a one-pot four-component, and efficient method by reaction of isatins, 1,3-indandione, diamine, and nitro ketene dithioacetal in the presence of Alum as a green catalyst for the synthesis of 4-nitro-2,3-dihydrospiro[imidazo[1,2-*a*]indeno[2,1-*e*]pyridine-5,3*'*-indoline]-2*'*,6(1*H*)-dione derivatives in good yields^32^.

## Results and discussion

**Figure 5:**
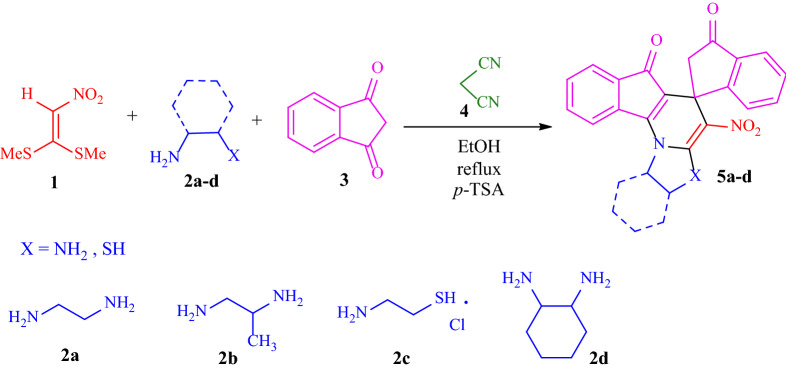
Synthetic outline for the formation of spiro[imidazo[1,2-*a*]indeno[2,1-*e*]pyridine-5,1*'*-indene **5**.

In this study, at first, a one-pot three-component reaction for the formation of spiro[imidazo[1,2-*a*]indeno[2,1-*e*]pyridine-5,1*'*-indene **5** is described through a reaction of 1,1-bis(methylthio)-2-nitroethene **1**, diamines/cysteamine hydrochloride **2**, and two units of 1,3-indandione **3** in the presence of malononitrile **4** as the promoter and *p*-TSA as the Brønsted-Lowry acid catalyst in one round-bottomed flask in ethanol as the reaction media under reflux conditions (Fig. 5). From the chemical structure of compound **5**, it can be seen that two units of 1,3-indanedione are in situ incorporated in the molecule and resulting in bindone that exhibits strong electron acceptor properties to heterocyclic ketene aminals (generated from the reaction of 1,1-bis(methylthio)-2-nitroethene **1** and diamines/cysteamine hydrochloride **2**) for synthesis the spiro imidazo-indeno pyridine compound.

**Figure 6:**
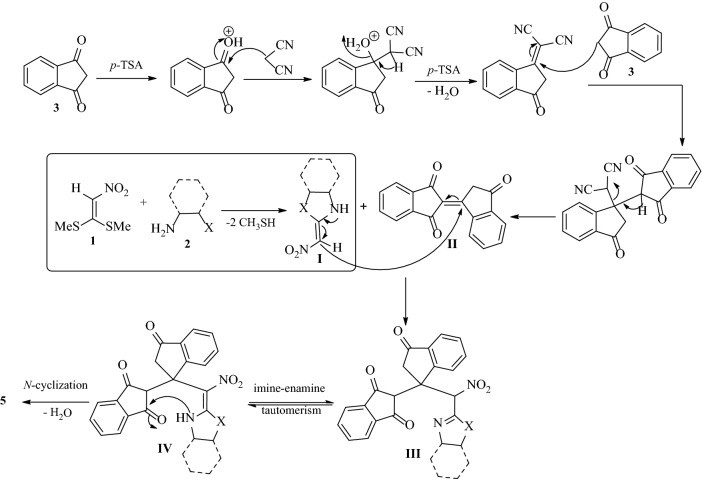
A plausible mechanism for the formation of **5**.

On the basis of the obtained results, a reaction mechanism for the synthesis of spiro[imidazo[1,2-*a*]indeno[2,1-*e*]pyridine-5,1*'*-indene **5**, is illustrated in Fig. 6. In order to form bindone **II**, it is possible that initially 1,3-indandione **3** is protonated in the presence of *p*-TSA. Then *Knoevenagel* condensation occurs between activated 1,3-indandione and malononitrile **4** as a promoter, and the second unit of 1,3-indandione **3** is added as an active CH compound to the *Knoevenagel* intermediate through aldol self-condensation which comes with the formation of the carbon–carbon double bond and removal of malononitrile and forms the bindone **II**. On the other, the formation of heterocyclic ketene aminal (HKA) **I** occurs through the addition of diamine **2** to 1,1-bis (methylthio)-2-nitroethene **1** with the removal of two thiomethyl groups. Then the HKA **I** as an enamine adds to the bindone **II** as a strong electron acceptor in a *Michael* addition to give open-chain intermediate **III**, which after successive imine-enamine tautomerization undergoes *N*-cyclization via attack of the secondary amino group to the more reactive carbonyl group of 1,3-indandione, give the product **5** (Fig. 6).

In the following, to prove the role of malononitrile as a promoter and the role of *p*-TSA as an acid catalyst for the formation of bindone, the reaction of 1,1-bis(methylthio)-2-nitroethene **1**, ethylenediamine **2a**, and two units of 1,3-indandione **3** was selected as a pattern for survey optimized reaction conditions (Table 1). The experiment began with the evaluation of the reaction without the use of malononitrile **4** as a promoter and without a catalyst in ethanol solvent under reflux conditions; no conversion happened within 24 h (entry 1). When the reaction was performed in the presence of *p*-TSA as the Brønsted-Lowry acid catalyst without the use of malononitrile **4** in ethanol under reflux conditions, the target compound did not form, and a number of spots were appeared on TLC (entry 2). In another attempt, the reaction was performed in the presence of **4** in the amount of 1.00 mmol without the use of the *p*-TSA in ethanol under reflux conditions, which again was not effective (entry 3). When the reaction was performed in the presence of **4** in the amount of 1.00 mmol as a promoter and *p*-TSA (20%mol) in ethanol under reflux conditions, the desired product **5a** was obtained as a red precipitate in 76% yield and in the time of 60 min (entry 4). The study followed by assessing the reaction using malononitrile **4** in the amount of 0.5 mmol in the same reaction condition; the target product was not achieved during 24 h (entry 5). In order to investigate the effect of the solvent on the reaction in the presence of **4** (1.00 mmol) and *p*-TSA (20%mol), the reaction was performed in polar solvents such as water (entry 6), and acetonitrile (entry 7) in reflux conditions for 24 h and the desired product did not form. In addition, the reaction was carried out in the well-known green solvent anhydrous ethanol diluted with one portion of water (entry 8). The results clearly indicate when used H_2_O/EtOH as a mixed solvent under reflux conditions, the target compound did not form. Choosing the proper solvent for this condensation is crucial. Among these polar solvents, ethanol as an inexpensive and environmentally friendly solvent can significantly improve product formation, reaction rate, and yields, and the use of malononitrile **4** as a promoter and *p*-TSA as an acid catalyst is essential (entry 4).

**Table 1:** Optimization of reaction conditions for the synthesis of **5a**.


Entry	Solvent	Compound 4 (eq)	Catalyst (20%mol)	Time (min)^a^	Yield (%)
1	EtOH	–	–	Overnight	NR^b^
2	EtOH	–	*p*-TSA	Overnight	No target compound
3	EtOH	(1 eq)	–	Overnight	NR^b^
**4**	**EtOH**	**(1 eq)**	***p*-TSA**	**60**	**76**
5	EtOH	(0.5 eq)	*p*-TSA	Overnight	No target compound
6	H_2_O	(1 eq)	*p*-TSA	Overnight	Intermediate II
7	CH_3_CN	(1 eq)	*p*-TSA	Overnight	No target compound
8	H_2_O/EtOH (1:1, v/v)	(1 eq)	*p*-TSA	Overnight	No target compound

Significant values are in bold.

General conditions: A mixture of 1,1-bis(methylthio)-2-nitro ethylene (1 mmol), ethylenediamine (1 mmol), 1,3-indandion (2 mmol) with or without using component **4** in various conditions.

^a^Reaction time is reported in accordance with the time of appearance of precipitate and thin-layer chromatography (TLC).

^b^Incomplete reaction with number of spots on TLC.

After optimizing the reaction, we surveyed the scope of these reactions by varying the derivatives of amine (ethylenediamine, 1,2-propanediamine, cysteamine, 1,2-diamino-cyclohexane **2a–d**) in the production of **5** which is shown in Table 2 (see Electronic Supplementary Material Fig. S1). The reaction proceeds cleanly and completely in the presence of different reagents to afford a library of spiro imidazo indeno pyridine products **5a–d** in 61–85% yields. It is noteworthy that products **5a–d** are novel compounds that have not been reported in the previous literature. We also used 1,3-diaminopropane, 1,4-diaminobutane, and ethanolamine as diverse amines to enhance the variety of the products structurally, but the reaction did not proceed.

**Table 2:** Synthesis of spiro[imidazo[1,2-*a*]indeno[2,1-*e*]pyridine-5,1*'*-indene (**5a–d**).

Entry	Diamine	Product	Time (h)	Yield (%)
1			1	76
2			2	72
3			6	85
4			7	61

**Figure 7:**
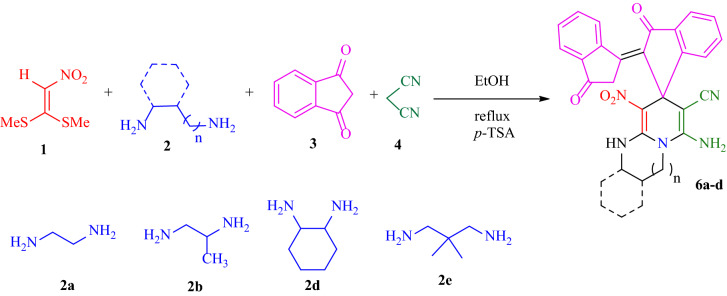
Synthetic outline for the formation of indenylidene-1*H*-spiro[imidazo[1,2-*a*]pyridine-7,1*'*-indene **6**.

In the continuation of the study, a new four-component reaction is developed for the formation of indenylidene-1*H*-spiro[imidazo[1,2-*a*]pyridine-7,1*'*-indene **6** via the reaction between 1,1-bis(methylthio)-2-nitroethene **1**, diamines 2, two units of 1,3-indandione **3,** and malononitrile **4** as one of the precursors in the presence of *p*-TSA (20%mol) as an acid catalyst in two steps (in two round-bottomed flasks) in ethanol as the reaction solvent under reflux conditions (Fig. 7). It is noteworthy that the synthesized final product contains active methylene and this active site can participate in more reactions to synthesize more complex compounds. The methylene group on the product structure is attached to two electron-withdrawing functional groups (including the carbonyl and *α*,*β*-unsaturated carbonyl groups), so is termed a reactive methylene group and the hydrogen atom can dissociate to give a stable anion due to resonance^33^. To verify that the product contains active methylene, we added product **6a** in potassium hydroxide solution in ethanol as a basic medium, and the product was dissolved, which is probably due to the removal of an *α*-hydrogen by the base that anion stabilized by resonance.

**Figure 8:**
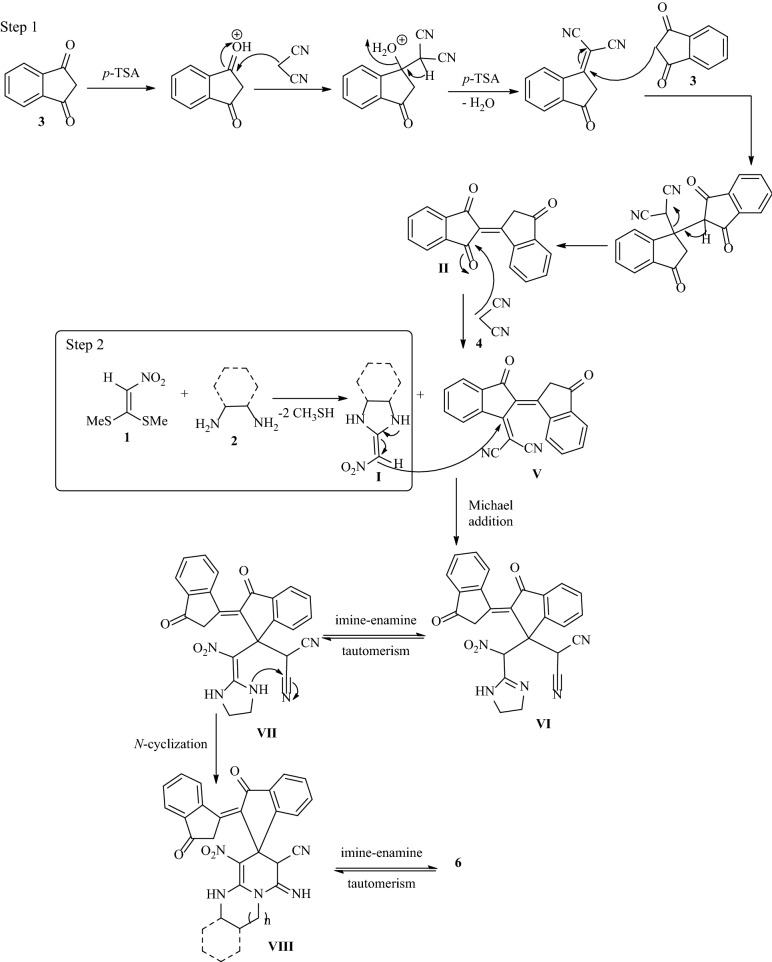
A plausible mechanism for the formation of **6**.

The acceptable reaction mechanism for the synthesis of indenylidene-1*H*-spiro[imidazo[1,2-*a*]pyridine-7,1'-indene **6** is designated in Fig. 8. The formation of bindone **II** proceeds similarly to the mechanism described in Fig. 6. Then *Knoevenagel* condensation occurs between bindone **II** and malononitrile **4**, which results in the formation of intermediate **V**. In the second step, the formation of (HKA) **I** occurs in a similar way with Fig. 6 and then added to the *Knoevenagel* intermediate **V** in a *Michael* addition type reaction to give open-chain intermediate **VI**. Intermediate **VI** is followed by successive imine-enamine tautomerization to form intermediate **VII**. Finally, intermediate **VII** undergoes intramolecular *N*-cyclization via an attack of the nitrogen to the nitrile group, and then imine-enamine tautomerization leads to the formation of the final product **6** (Fig. 8).

As shown in Table 3, we studied the structural diversity of this reaction using diamines **2** in the production of **6a-d** (see Electronic Supplementary Material Fig. S1). Compounds named indenylidene-1*H*-spiro[imidazo[1,2-*a*]pyridine-7,1'-indene were obtained in moderate to high yields (62–98%) with an almost long reaction time (24–32 h). We also used 2 equivalents of primary amines such as ethylamine, and propylamine to increase the structural diversity of the products, but the reaction did not occur.

**Table 3:** Synthesis of indenylidene-1*H*-spiro[imidazo[1,2-*a*]pyridine-7,1′-indene (**6a–d**).

Entry	Diamine	Product	Time (h)	Yield (%)
1			24	98
2			28	98
3			24	62
4			32	82

**Figure 9:**
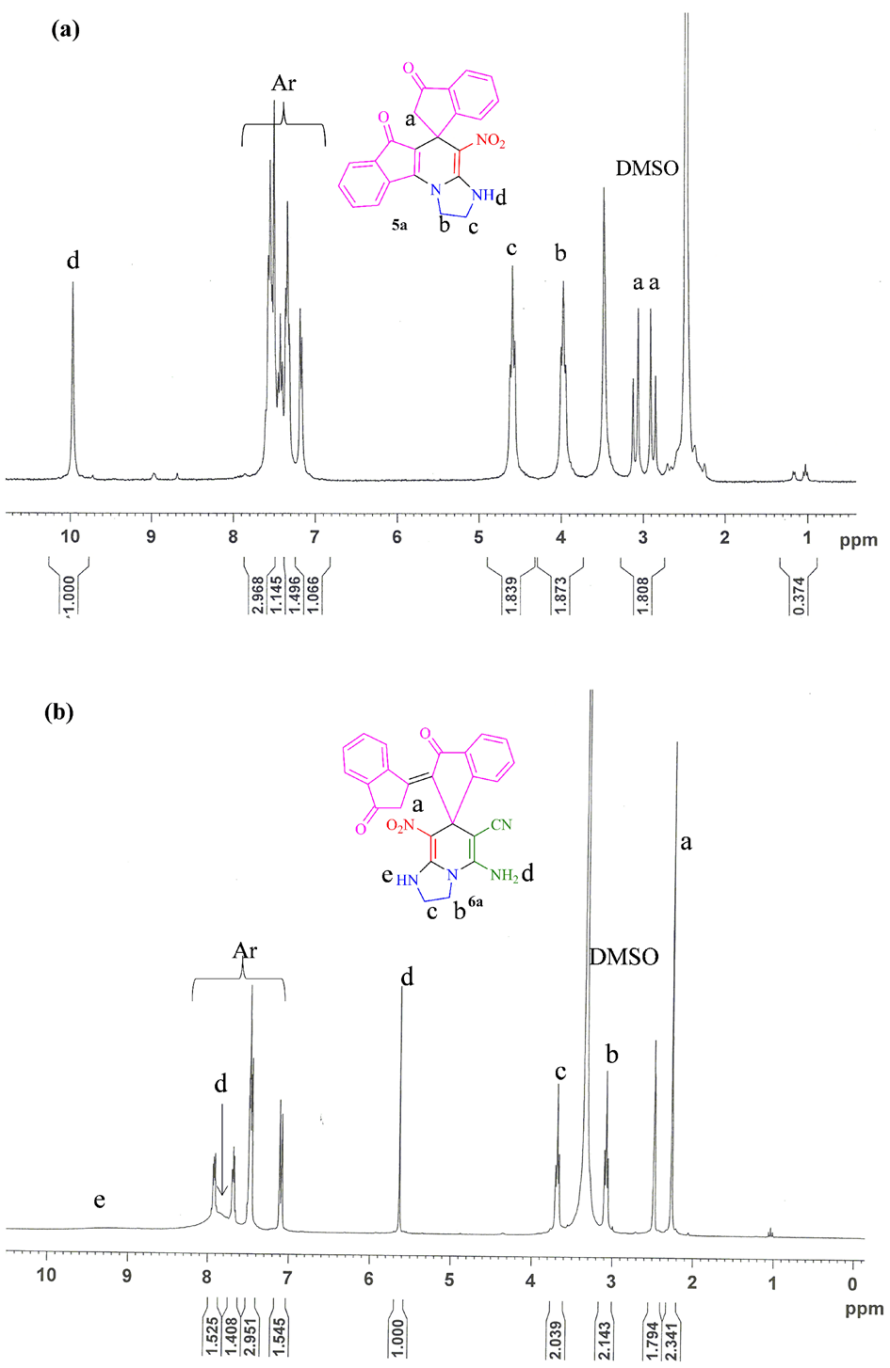
The ^1^H NMR spectra of (**a**) **5a** and (**b**) **6a**.

The structures of the synthesized products were deduced from their IR, mass spectrometry, ^1^H and ^13^C NMR spectra. The mass spectra of **5a** displayed molecular ion peaks at 385 m*/z* values, which were in agreement with the proposed structures. In the ^1^H NMR spectrum of **5a** in DMSO-*d*_*6*_, because diastereotopic hydrogens have different chemical shifts, they can undergo spin–spin coupling to each other and two doublet peaks were observed for two protons of methylene group, (*δ* 2.88 ppm, ^2^*J*_HH_ = 18.6 Hz) for one proton and (*δ* 3.09 ppm, ^2^*J*_HH_ = 18.3 Hz) for another proton of methylene group. Also, the ^1^H NMR spectrum of **5a** showed two triplets for the two methylene groups, CH_2_NH and CH_2_N (*δ* 3.98 ppm, ^3^*J*_HH_ = 8.7 Hz) and (*δ* 4.60 ppm, ^3^*J*_HH_ = 8.7 Hz) respectively, aromatic region of the spectrum (*δ* 7.17–7.72 ppm) for the aromatic moieties, and one broad singlet for the NH group (*δ* 9.97 ppm, D_2_O exchangeable) (Fig. 9a). The ^1^H decoupled ^13^C NMR spectrum of **5a** showed 20 distinct signals in agreement with proposed structure. Two peaks at *δ* 189.4 and 204.6 ppm, which were specified as two carbonyl groups and the specific peaks of C_spiro_, C–NO_2_, C=**C**–CO, **C**=C–CO and **C**=C–NO_2_, were assigned at *δ* 44.7, 109.5, 112.1, 153.2 and 158.2 ppm respectively, verified the selective synthesized of **5a** (See, Electronic Supplementary Material Fig. S2a–e). When 1,2-propanediamine was used, it resulted in product **5b**, which was characterized by the ^1^H and ^13^C NMR spectra (See, Electronic Supplementary Material Fig. S3a,b). When cysteamine hydrochloride was used, it resulted in product **5c**, which was characterized by the ^1^H, D_2_O exchange and ^13^C NMR, IR and Mass spectra (See, Electronic Supplementary Material Fig. S4a–e). Derivation of compound **5** with 1,2-diamino-cyclohexane yielded the corresponding product **5d** (See, Electronic Supplementary Material Fig. S5a).

The ^1^H NMR spectrum of **6a** displayed one singlet for CH_2_ group (*δ* 2.27 ppm), two triplets for CH_2_N and CH_2_NH groups (*δ* 3.08 ppm, ^3^*J*_HH_ = 6.6 Hz) and (*δ* 3.69 ppm, ^3^*J*_HH_ = 6.3 Hz) respectively, one singlet for one proton of NH_2_ group (*δ* 5.64 ppm, D_2_O exchangeable), aromatic region of the spectrum for the aromatic moieties (*δ* 7.10–7.93 ppm), one broad singlet for another proton of NH_2_ (*δ* 7.85 ppm, D_2_O exchangeable) and one broad singlet for NH group of imidazole (*δ* 9.34 ppm, D_2_O exchangeable) (Fig. 9b) (See, Electronic Supplementary Material Fig. S6a–e). Derivation of compound **6** with 1,2-propanediamine yielded the corresponding product **6b** which was characterized by the ^1^H and ^13^C NMR, IR and Mass spectra (See, Electronic Supplementary Material Fig. S7a–d). When 1,2-diamino-cyclohexane was used, it resulted in product **6c**, which was characterized by the ^1^H and ^13^C NMR, IR and Mass spectra (See, Electronic Supplementary Material Fig. S8a–d). Eventually, the derivation of compound **6** with 2,2-dimethyl-1,3-propanediamine yielded the corresponding product **6d** (See, Electronic Supplementary Material Fig. S9a,b). In addition, the structure of intermediate **II** was characterized by the ^1^H NMR spectrum (see Electronic Supplementary Material Fig. S2).

## Conclusion

An acid-promoted annulation reaction of bindone with heterocyclic ketene aminals showed very interesting molecular diversity under different reaction conditions. This reaction provided a convenient protocol for selective synthesis of complex bindone-fused spiro polycyclic compounds. Indeed, the progress of the acid-promoted annulation in EtOH at one or two steps process selectively gave different spiro compounds. A comprehensive reaction mechanism is rationally proposed in the form of sequential self-aldol condensation/*Knoevenagel* condensation/*Michael* addition/imine-enamine tautomerism/intramolecular *N*-cyclization sequences. The advantages of this reaction included convenient operation, accessible raw materials, easy workup, and good yields, which results in the synthesis of biologically and chemically valuable indanone-containing compounds. It is noteworthy that bindone and the synthesized final product contain active methylene and this active site can participate in more reactions to synthesize more complex compounds. Further investigation of these reactions is currently underway in our laboratory and will be reported in due course.

## Experimental

### General

The 1,1-bis(methylthio)-2-nitroethene, different diamines, cysteamine hydrochloride, 1,3-indandione**,** malononitrile, *p*-TSA and solvents were obtained from Sigma Aldrich and Fluka Co. used without further purification. IR spectra: Bruker Tensor 27 spectrometer. NMR spectra: Bruker DRX-300 Avance instrument (300 MHz for ^1^H and 75.4 MHz for ^13^C) with DMSO-*d*_6_ as solvents. Chemical shifts are expressed in parts per million (ppm), and coupling constant (*J*) are reported in hertz (Hz). Mass spectra: Agilent 5975C VL MSD with Triple-Axis detector operating at an ionization potential of 70 eV. Elemental analyses were performed using a PerkinElmer 2004 series [II] CHN elemental analyzer.

### General procedure for the formation of 5a-d

A mixture of diamine (1 mmol), 1,1-bis(methylthio)-2-nitro ethylene (0.165 g, 1 mmol), and 10 mL EtOH in a 50 mL round-bottomed flask fitted with a reflux condenser and was heated with stirring in an oil-bath at reflux temperature for 4 h^34^, after that 1,3-indandion (2 mmol), malononitrile (1 mmol) and *p*-TSA (20%mol) were added to the reaction mixture, and it was refluxed for a period of time shown in Table 2, which monitored by TLC, ethyl acetate/*n*-hexane, 1:1. Then, the reaction mixture was cooled to room temperature and the precipitate was filtered to give the crude product. The solid was washed with 96% ethanol and dried in an oven in 150 °C to give product **5** and analyzed by ^1^H NMR and ^13^C NMR. Only in the case of **5c**, triethylamine (139 µL, 1 mmol) was added at the first step for the release of cysteamine salt.

### General procedure for the formation of 6a-d

The first step of HKA formation was performed similarly to the synthesis method of **5**^34^. In another 50 mL round-bottomed flask, the mixtures of 1,3-indandion (2 mmol), malononitrile (1 mmol), *p*-TSA (20%mol) and 5 mL EtOH were added and the reaction mixture was stirred for 1 h at room temperature. Next, two round-bottomed flasks were added and the solution was refluxed for the time given in Table 3. The progress of the reaction was monitored by TLC using ethyl acetate/*n*-hexane (1:1). After completion of the reaction, the precipitated product was filtered off and washed on the filter funnel with a small amount of EtOH to give pure products **6a–d**.

#### 4-Nitro-2,3-dihydro-1H,6H-spiro[imidazo[1,2-a]indeno[2,1-e]pyridine-5,1'-indene]-3',6(2'H)-dione (5a)

Red solid; yield: 0.293 g (76%); mp 308–310 °C; IR (KBr) (ῡ_max_): 3419 (NH), 2922 (C–H), 1723 (C=O), 1640 (C=O), 1456, 1377 (NO_2_), 1214 (C–N), 772 cm^−1^; ^1^H NMR (300 MHz, DMSO-*d*_*6*_): δ = 2.88 (1H, d, ^2^*J*_HH_ = 18.6 Hz, CH_2_), 3.09 (1H, d, ^2^*J*_HH_ = 18.3 Hz, CH_2_), 3.98 (2H, t, ^3^*J*_HH_ = 8.7 Hz, CH_2_), 4.60 (2H, t, ^3^*J*_HH_ = 8.7 Hz, CH_2_N), 7.17–7.72 (8H, m, ArH), 9.97 (1H, s, NH, D_2_O exchange); ^13^C NMR (75 MHz, DMSO-*d*_*6*_): δ = 43.4 (CH_2_), 44.7 (C_spiro_), 46.3 (CH_2_NH), 49.6 (CH_2_N), 109.5 (C–NO_2_), 112.1 (CO–**C**=C), 121.8, 121.9, 125.1, 128.0, 131.3, 132.9, 133.4, 135.2, 137.3, 152.4 (Ar), 153.2 (CO**–**C=**C**), 158.2 (**C**=C–NO_2_), 189.4 (CO), 204.6 (CO); MS (EI, 70 eV): m/z (%) = 385 (1) [M]^+^, 338 (57), 294 (73), 216 (100), 91 (42).

#### 2-Methyl-4-nitro-2,3-dihydro-1H,6H-spiro[imidazo[1,2-a]indeno[2,1-e]pyridine-5,1′-indene]-3′,6(2′H)-dione (5b)

Red solid; yield: 0.287 g (72%); mp 301–304 °C; ^1^H NMR (300 MHz, DMSO-*d*_*6*_): δ = 1.47 (3H, d, ^3^*J*_HH_ = 6.8 Hz, CH_3_), 2.70 (1H, d, ^2^*J*_HH_ = 18.0 Hz, CH_2_), 2.87 (1H, d, ^2^*J*_HH_ = 18.0 Hz, CH_2_), 4.12–4.19 (1H, m, CH), 4.31–4.48 (1H, m, CH_2_N), 4.60–4.76 (1H, m, CH_2_N), 7.17–7.94 (8H, m, ArH), 10.09 (1H, br s, NH); ^13^C NMR (75 MHz, DMSO- *d*_*6*_): δ = 21.1 (CH_3_), 43.4 (CH_2_), 49.7 (C_spiro_), 52.9 (CH_2_NH), 53.0 (CH_2_N), 109.1 (C–NO_2_), 112.2 (CO–**C**=C), 121.7, 121.9, 125.1, 125.2, 127.9, 131.2, 132.8, 134.9, 135.3, 137.3, 152.3 (Ar), 152.4 (CO**–**C=**C**), 158.3 (**C**=C–NO_2_), 189.4 (CO), 204.4 (CO).

#### 4′-Nitro-1′,2′-dihydro-6'H-spiro[indene-1,5′-indeno[2,1-e]thiazolo[3,2-a]pyridine]-3,6′(2H)-dione (5c)

Red solid; yield: 0.341 g (85%); mp 313–316 °C; IR (KBr) (ῡ_max_): 1700 (C=O), 1621 (C=O), 1520 (NO_2_), 1269 (C–N), 758 cm^−1^; ^1^H NMR (300 MHz, DMSO-*d*_*6*_): δ = 2.62 (1H, d, ^2^*J*_HH_ = 16.2 Hz, CH_2_), 3.08 (1H, d, ^2^*J*_HH_ = 16.2 Hz, CH_2_), 3.46 (2H, t, ^3^*J*_HH_ = 7.4 Hz, CH_2_), 4.74 (2H, t, ^3^*J*_HH_ = 7.8 Hz, CH_2_N), 7.12–7.63 (8H, m, ArH); ^13^C NMR (75 MHz, DMSO-*d*_*6*_): δ = 29.3 (CH_2_S), 43.9 (CH_2_), 50.5 (C_spiro_), 52.9 (CH_2_N), 114.8 (CO–**C**=C), 122.0 (C–NO_2_), 122.3, 123.0, 125.6, 126.1, 128.6, 131.2, 132.9, 133.3, 135.4, 135.7, 151.9 (Ar), 157.6 (**C**=C–NO_2_), 161.1 (CO**–**C=**C**), 190.0 (CO), 203.9 (CO); MS (EI, 70 eV): m/z (%) = 402 (6) [M]^+^, 384 (16), 356 (6), 322 (100), 294 (9), 267 (12), 238 (14), 105 (5), 76 (2).

#### 6-Nitro-1,3,4,4a,5,13a-hexahydro-2H,8H-spiro[benzo[4,5]imidazo[1,2-a]indeno[2,1-e]pyridine-7,1′-indene]-3′,8(2′H)-dione (5d)

Red solid; yield: 0.267 g (61%); mp 320–324 °C; ^1^H NMR (300 MHz, DMSO-*d*_*6*_): δ = 1.30–1.55 (2H, m, CH_2_), 1.56–1.78 (2H, m, CH_2_), 2.04–2.27 (4H, m, 2CH_2_), 2.64 (1H, d, ^2^*J*_HH_ = 18.2 Hz, CH_2_), 2.89 (1H, d, ^2^*J*_HH_ = 18.2 Hz, CH_2_), 4.29–4.37 (1H, m, CH), 5.03–5.14 (1H, m, CHN), 7.14–7.79 (8H, m, ArH), 9.86 (1H, br s, NH).

#### (Z)-5-Amino-8-nitro-3′-oxo-2′-(3-oxo-2,3-dihydro-1H-inden-1-ylidene)-2,2′,3,3′-tetrahydro-1H-spiro[imidazo[1,2-a]pyridine-7,1′-indene]-6-carbonitrile (6a)

Brown solid; yield: 0.440 g (98%); mp 309 °C; IR (KBr) (ῡ_max_): 3415, 3277 (NH_2_, NH), 2924 (C–H), 2209 (CN), 1614 (C=O), 1527 (C=C), 1384, 1451 (NO_2_), 1257 (C–N), 756 cm^−1^; ^1^H NMR (300 MHz, DMSO-*d*_*6*_): δ = 2.27 (2H, s, CH_2_), 3.08 (2H, t, ^3^*J*_HH_ = 6.6 Hz, CH_2_), 3.69 (2H, t, ^3^*J*_HH_ = 6.3 Hz, CH_2_N), 5.64 (1H, s, NH, D_2_O exchange), 7.10 (2H, d, ^3^*J*_HH_ = 7.8 Hz, ArH), 7.46–7.51 (2H, m, ArH), 7.66–7.70 (2H, m, ArH), 7.85 (1H, br s, NH, D_2_O exchange), 7.93 (2H, d, ^3^*J*_HH_ = 8.1 Hz, ArH), 9.34 (1H, br s, NH, D_2_O exchange); ^13^C NMR (75 MHz, DMSO-*d*_*6*_): δ = 21.2 (CH_2_), 38.5 (CH_2_NH), 42.9 (CH_2_N), 57.8 (C_spiro_), **C**–CN (92.6), 116.8 (C–NO_2_), 121.2 (CN), 122.7, 125.9, 128.5, 128.6, 131.3, 131.4, 136.1, 136.6 (Ar), 138.3 (CO–**C**=C), 145.9 (CO**–**C=**C**), 162.8 (**C**=C–NO_2_), 165.0 (C-NH_2_), 192.0 (CO), 199.2 (CO); MS (EI, 70 eV): m/z (%) = 451 (0.5) [M]^+^, 236 (16), 207 (56), 172 (91), 91 (100), 65 (34).

#### (Z)-5-Amino-2-methyl-8-nitro-3′-oxo-2′-(3-oxo-2,3-dihydro-1H-inden-1-ylidene)-2,2′,3,3′-tetrahydro-1H-spiro[imidazo[1,2-a]pyridine-7,1′-indene]-6-carbonitrile (6b)

Brown solid; yield: 0.455 g (98%); mp 282–286 °C; IR (KBr) (ῡ_max_): 3485, 3414 (NH, NH_2_), 2865 (C–H), 2202 (CN), 1623 (C=O), 1541 (C=O), 1467, 1380 (NO_2_), 1177 (C–N), 620 cm^−1^; ^1^H NMR (300 MHz, DMSO-*d*_*6*_): δ = 1.20 (3H, d, ^3^*J*_HH_ = 7.8 Hz, CH_3_), 2.28 (2H, s, CH_2_), 3.43–3.53 (1H, m, CH), 3.62 (2H, d, ^3^*J*_HH_ = 6.6 Hz, CH_2_N), 5.69 (1H, s, NH), 7.05 (2H, d, ^3^*J*_HH_ = 7.8 Hz, ArH), 7.46–7.51 (2H, m, ArH), 7.72 (2H, d, ^3^*J*_HH_ = 6.9 Hz, CH_2_N), 7.91 (1H, br s, NH), 7.91–7.94 (2H, m, ArH), 9.31 (1H, br s, NH); ^13^C NMR (75 MHz, DMSO-*d*_*6*_): δ = 16.7 (CH_3_), 21.2 (CH_2_), 46.7 (CH), 48.3 (CH_2_N), 57.9 (C_spiro_), 92.7 (**C**–CN), 116.87 (C-NO_2_), 121.3 (CN), 121.5, 122.6, 125.9, 128.7, 131.3, 131.4, 136.0, 136.6, 138.3 (Ar), 145.3 (CO–**C**=C), 162.1 (CO**–**C=**C**), 163.0 (**C**=C–NO_2_), 165.0 (C–NH_2_). MS (EI, 70 eV): m/z (%) = 465 (0.3) [M]^+^, 370 (4), 322 (12), 250 (24), 221 (42), 172 (58), 91 (69), 44 (100).

#### (Z)-1-Amino-4-nitro-3′-oxo-2′-(3-oxo-2,3-dihydro-1H-inden-1-ylidene)-2′,3′,5a,6,7,8,9,9a-octahydro-5H-spiro[benzo[4,5]imidazo[1,2-a]pyridine-3,1′-indene]-2-carbonitrile (6c)

Brown solid; yield: 0.314 g (62%); mp 284–288 °C; IR (KBr) (ῡ_max_): 3432, 3265 (NH, NH_2_), 2925 (C–H), 2208 (CN), 1609 (C=O), 1509 (C=O), 1446, 1384 (NO_2_), 1202 (C–N), 755 (Ar) cm^−1^; ^1^H NMR (300 MHz, DMSO-*d*_*6*_): δ = 1.28–1.51 (4H, m, 2CH_2_), 1.61–2.06 (4H, m, 2CH_2_), 2.27 (2H, s, CH_2_), 3.39–3.52 (1H, m, CH), 4.08–4.16 (1H, m, CHN), 5.76 (1H, d, ^3^*J*_HH_ = 7.8 Hz, NH), 7.09 (1H, d, ^3^*J*_HH_ = 7.8 Hz, ArH), 7.37–7.89 (7H, m, ArH), 8.52 (1H, br s, NH), 9.13 (1H, br s, NH); ^13^C NMR (75 MHz, DMSO-*d*_*6*_): δ = 20.2 (2CH_2_), 26.0 (CH_2_), 27.5 (CH_2_), 50.4 (CH_2_), 54.0 (CH), 57.2 (CH), 58.3 (C_spiro_), 92.7 (**C**–CN), 93.7 (C–NO_2_), 116.7 (CN), 122.5, 125.8, 125.9, 128.5, 128.6, 131.1, 131.3, 131.4, 136.9, 138.2 (Ar), 146.0 (CO–**C**=C), 162.4 (CO**–**C=**C**), 162.5 (**C**=C–NO_2_), 165.1 (C–NH_2_). MS (EI, 70 eV): m/z (%) = 505 (0.2) [M]^+^, 370 (14), 322 (24), 224 (81), 195 (46), 172 (83), 91 (100), 65 (38).

#### (Z)-6′-Amino-3′,3′-dimethyl-9′-nitro-3-oxo-2-(3-oxo-2,3-dihydro-1H-inden-1-ylidene)-1′,2,2′,3,3′,4′-hexahydrospiro[indene-1,8′-pyrido[1,2-a]pyrimidine]-7′-carbonitrile (6d)

Brown solid; yield: 0.404 g (82%); mp 280–281 °C; IR (KBr) (ῡ_max_): 3434, 3349 (NH, NH_2_), 2927 (C–H), 2207 (CN), 1618 (C=O), 1574 (C=O), 1440, 1387 (NO_2_), 1174 (C–N), 749 (Ar) cm^−1^; ^1^H NMR (300 MHz, DMSO-*d*_*6*_): δ = 0.97 (6H, s, 2CH_3_), 2.27 (2H, s, CH_2_), 2.78 (4H, s, 2CH_2_), 5.63 (1H, s, NH), 7.11 (2H, d, ^3^*J*_HH_ = 7.5 Hz, ArH), 7.58 (2H, d, ^3^*J*_HH_ = 7.5 Hz, ArH), 7.75–7.84 (4H, m, ArH), 7.87 (1H, br s, NH), 9.07 (1H, br s, NH).

## Supplementary Information

Supplementary Information.

## Data Availability

All data generated or analysed during this study are included in this published article [and its Supplementary Information file as the ‘Supplementary Material’ file].
