# Supplementary material for: Synthesis of diverse spiro-imidazo pyridine-indene derivatives via acid-promoted annulation reaction of bindone and heterocyclic ketene aminals
Source: Sci Rep. 2022 Jul 22;12:12550. doi: 10.1038/s41598-022-16959-w (PMC9307797; doi:10.1038/s41598-022-16959-w)
Supplement: Supplementary file 1 — Supplementary Information. [file 41598_2022_16959_MOESM1_ESM.doc]

**Supporting Information**

**Synthesis of Diverse Spiro-imidazo Pyridine-indene Derivatives *via* Acid-Promoted Annulation Reaction of Bindone and Heterocyclic Ketene Aminals**

Shima Nasri, Mohammad Bayat,^*^ Fatemeh Rostami Miankooshki

*Department of Chemistry, Faculty of Science, Imam Khomeini International University, Qazvin, Iran*

*E-mail:* [*m.bayat@sci.ikiu.ac.ir*](mailto:m.bayat@sci.ikiu.ac.ir)*;* [*bayat_mo@yahoo.com*](mailto:bayat_mo@yahoo.com)

**The Table of Contents**

| **Title** | **Page** |
| --- | --- |
| Title, author’s name, address and table of contents | 1 |
| General remarks and **FIGURE 1**. | 2 |
| ^1^H and D_2_O exchange and ^13^C NMR and IR and Mass spectra of **5a** | 3-7 |
| ^1^H and ^13^C NMR spectra of **5b** | 8-9 |
| ^1^H and D_2_O exchange and ^13^C NMR and IR and Mass spectra of **5c** | 10-14 |
| ^1^H NMR spectrum of **5d** | 15 |
| ^1^H and D_2_O exchange and ^13^C NMR and IR and Mass spectra of **6a** | 16-20 |
| ^1^H and ^13^C NMR and IR and Mass spectra of **6b** | 21-24 |
| ^1^H and ^13^C NMR and IR and Mass spectra of **6c** | 25-28 |
| ^1^H NMR and IR spectra of **6d** | 29-30 |
| ^1^H NMR of intermediate **II** | 31 |

**Experimental Section**

**General remarks:**

The 1,1-bis(methylthio)-2-nitroethene, different diamines, cysteamine hydrochloride, 1,3-indandione**,** malononitrile, *p*-TSA and solvents were obtained from Sigma Aldrich and Fluka Co. used without further purification. IR spectra: Bruker Tensor 27 spectrometer. NMR spectra: Bruker DRX-300 Avance instrument (300 MHz for ^1^H and 75.4 MHz for ^13^C) with DMSO-*d*_6_ as solvents. Chemical shifts are expressed in parts per million (ppm), and coupling constant (*J*) are reported in hertz (Hz). Mass spectra: Agilent 5975C VL MSD with Triple-Axis detector operating at an ionization potential of 70 eV. Elemental analyses were performed using a PerkinElmer 2004 series [II] CHN elemental analyzer.

**Fig. S1**. Structure of all products **5a-d** and **6a-d**.

**
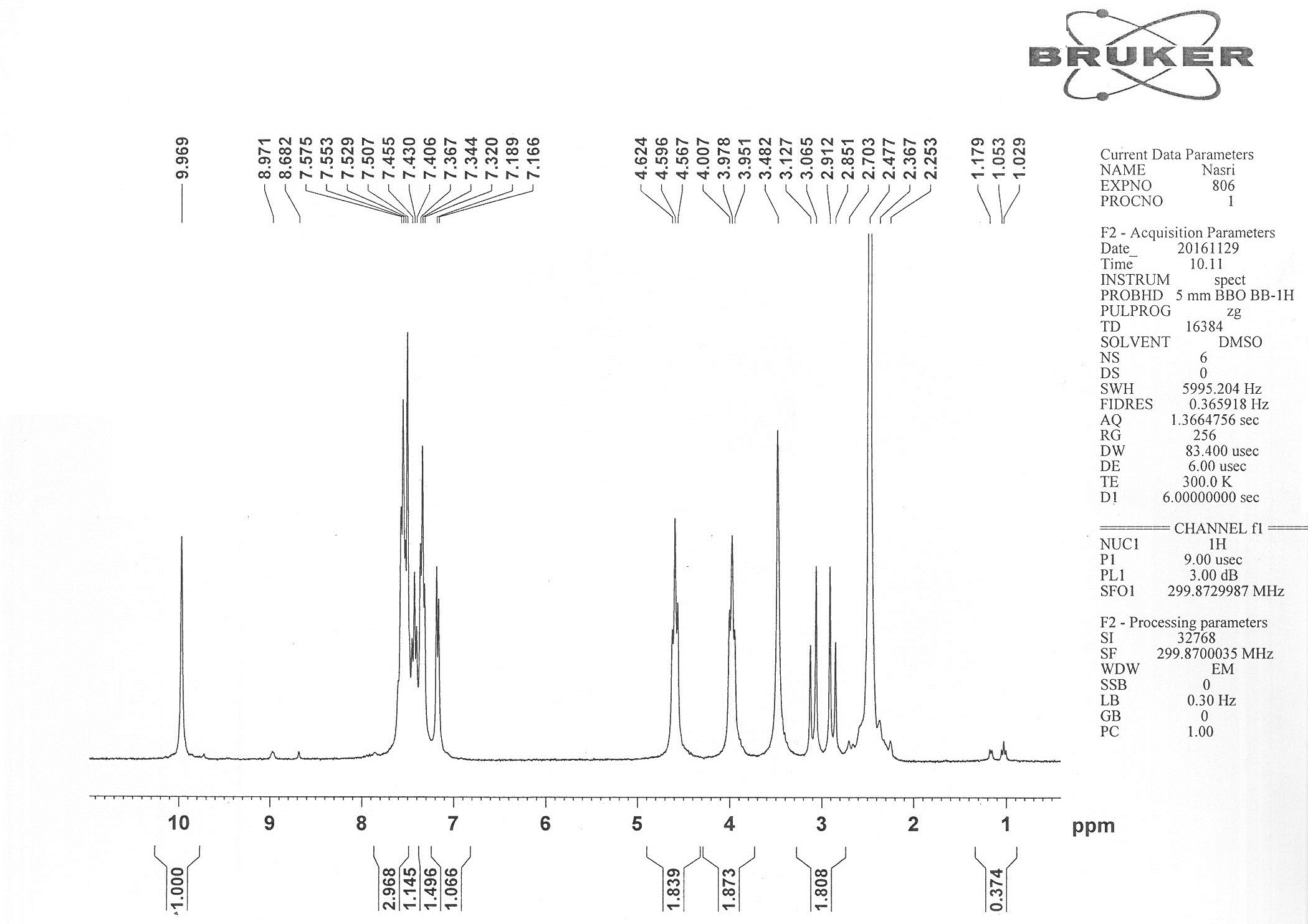
**

**Fig. S2a.** **^1^H NMR of 5a**


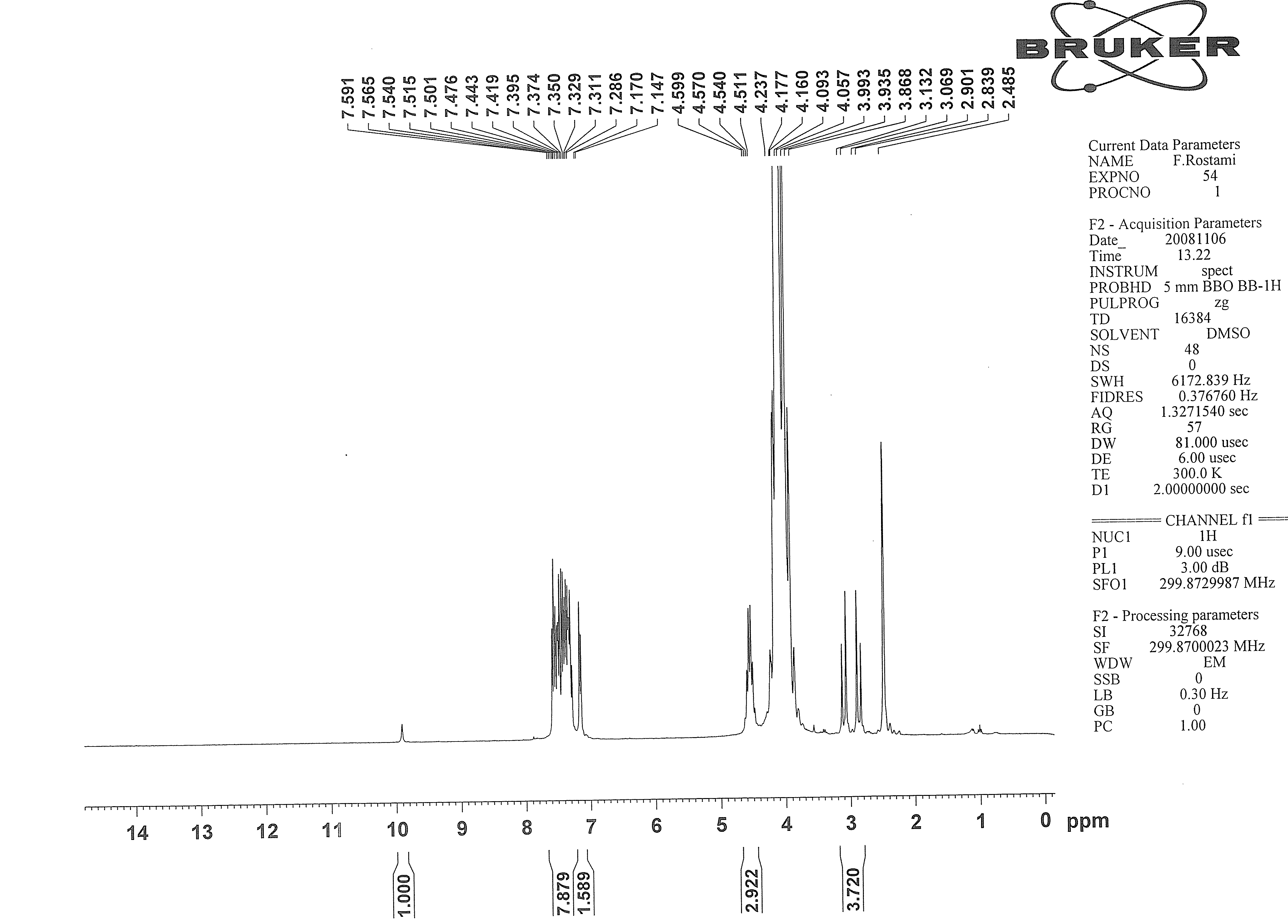


**Fig. S2b. D_2_O exchange of 5a**


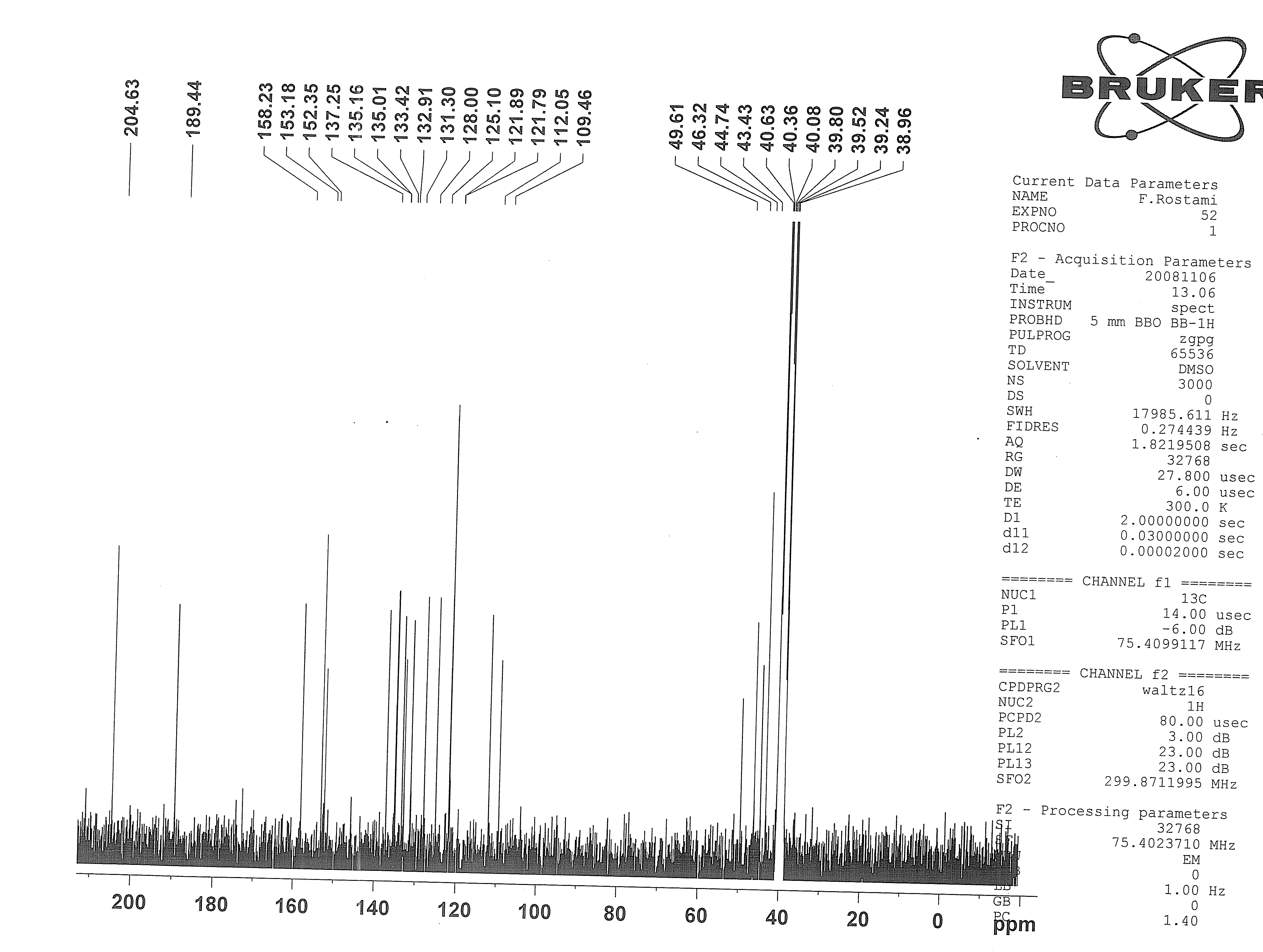


**Fig. S2c. ^13^C NMR of 5a**


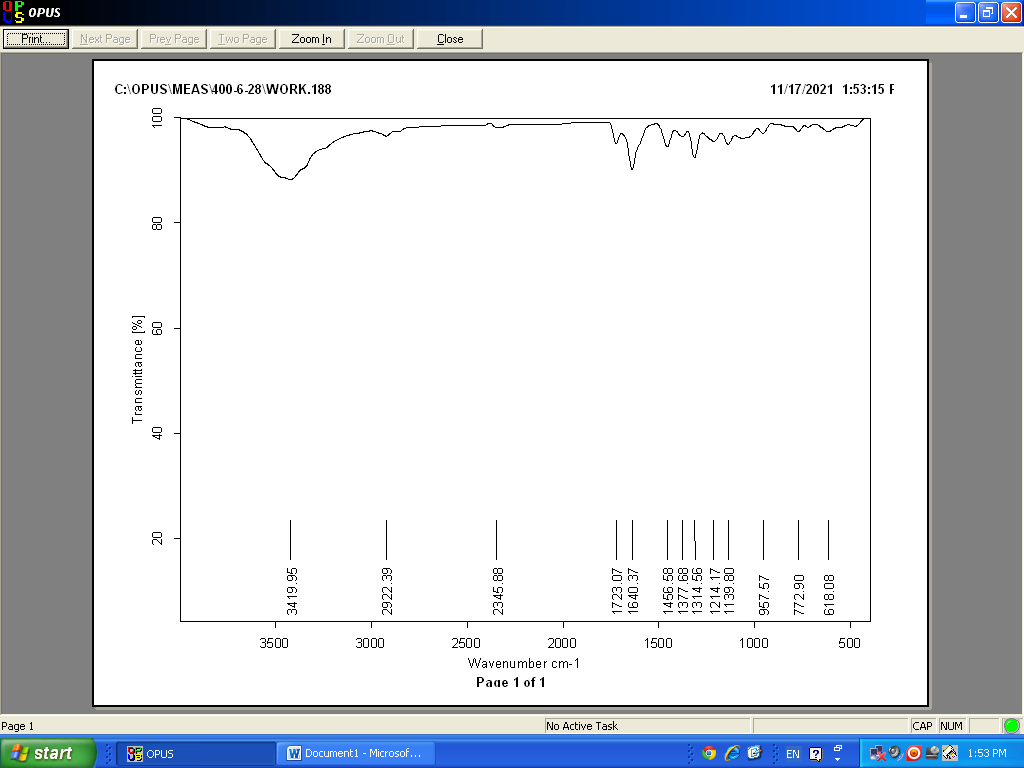


**Fig. S2d. IR of 5a**

**Fig. S2e. Mass of 5a**


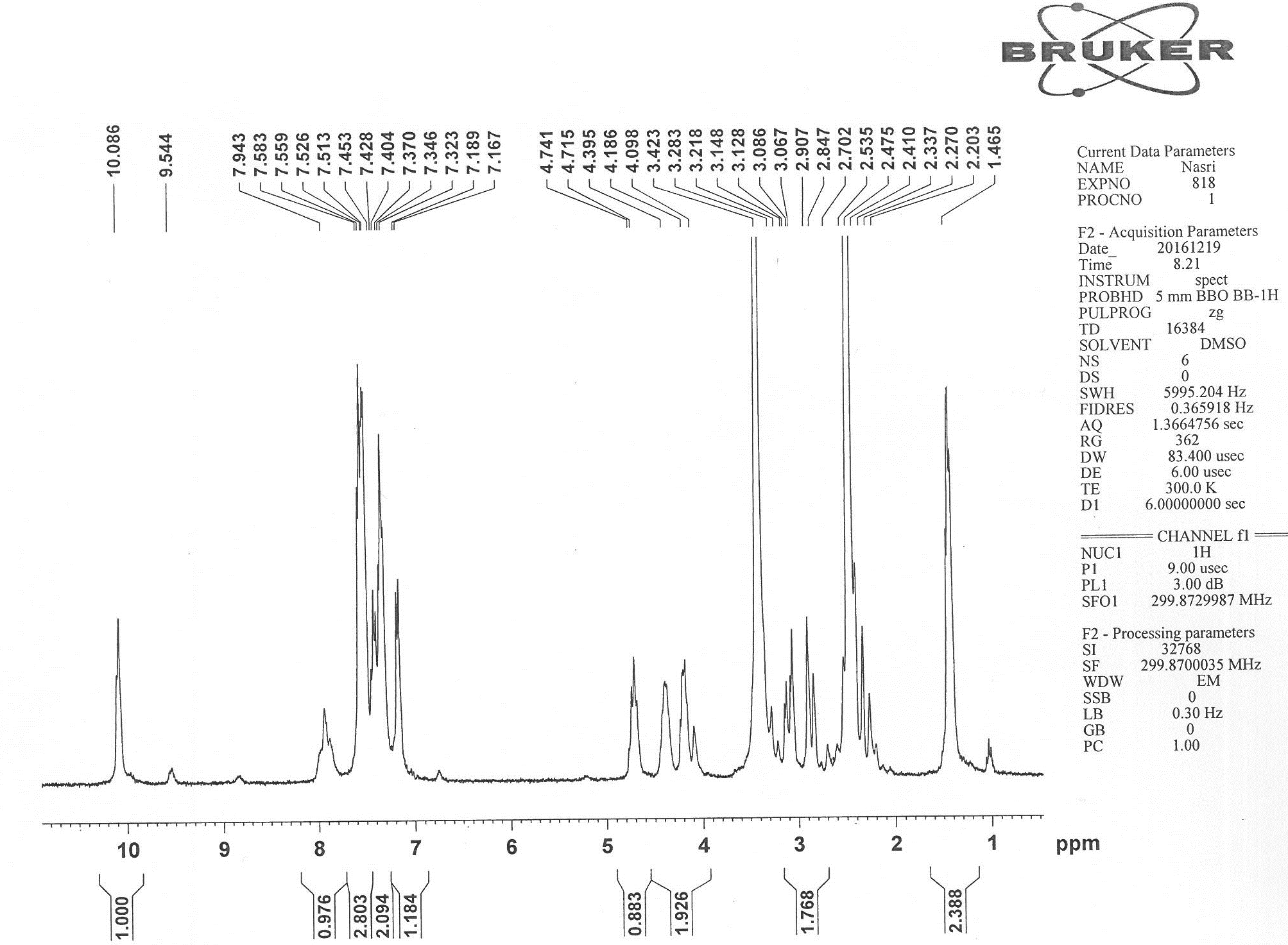


**Fig. S3a. ^1^H NMR of 5b**


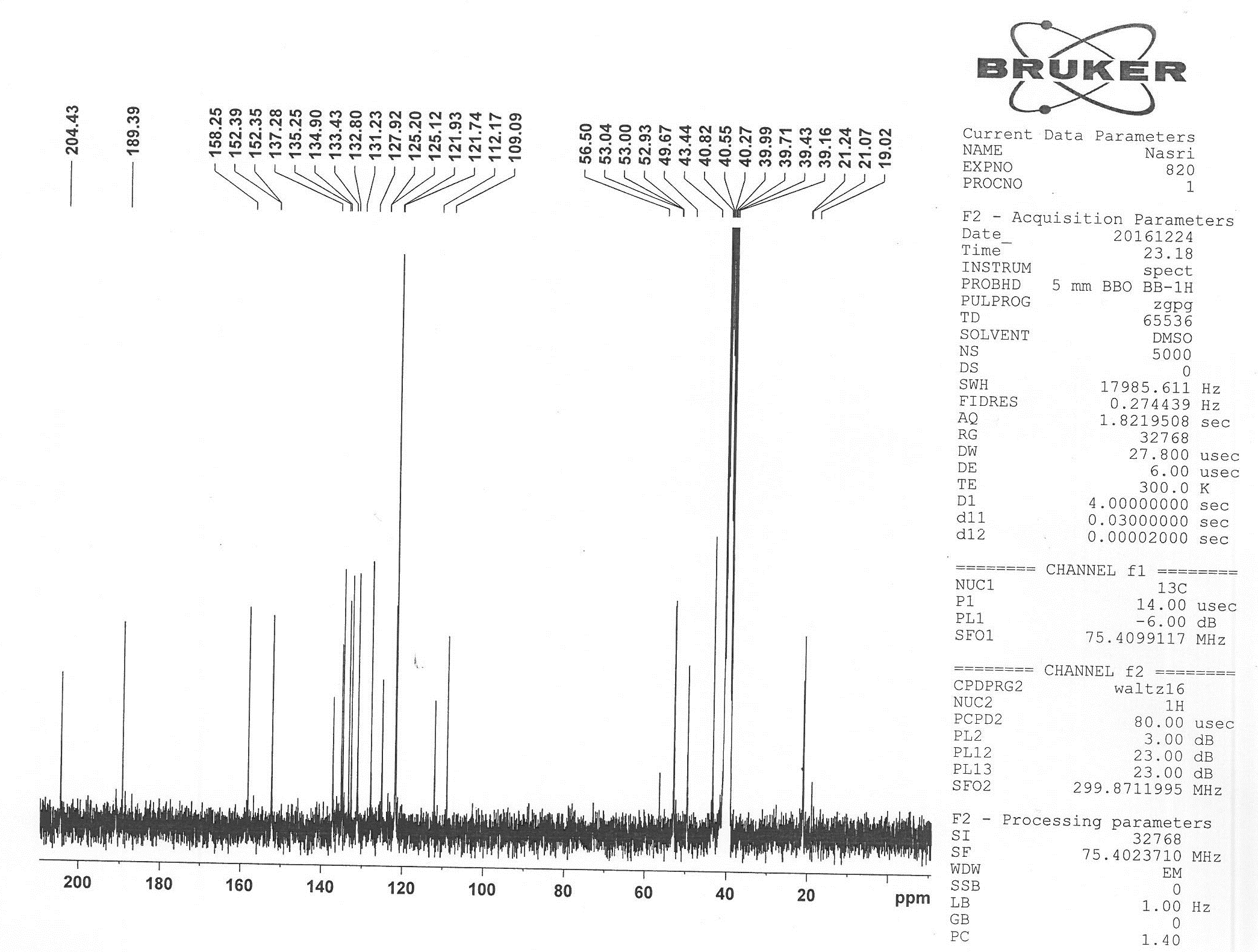


**Fig. S3b. ^13^C NMR of 5b**

**
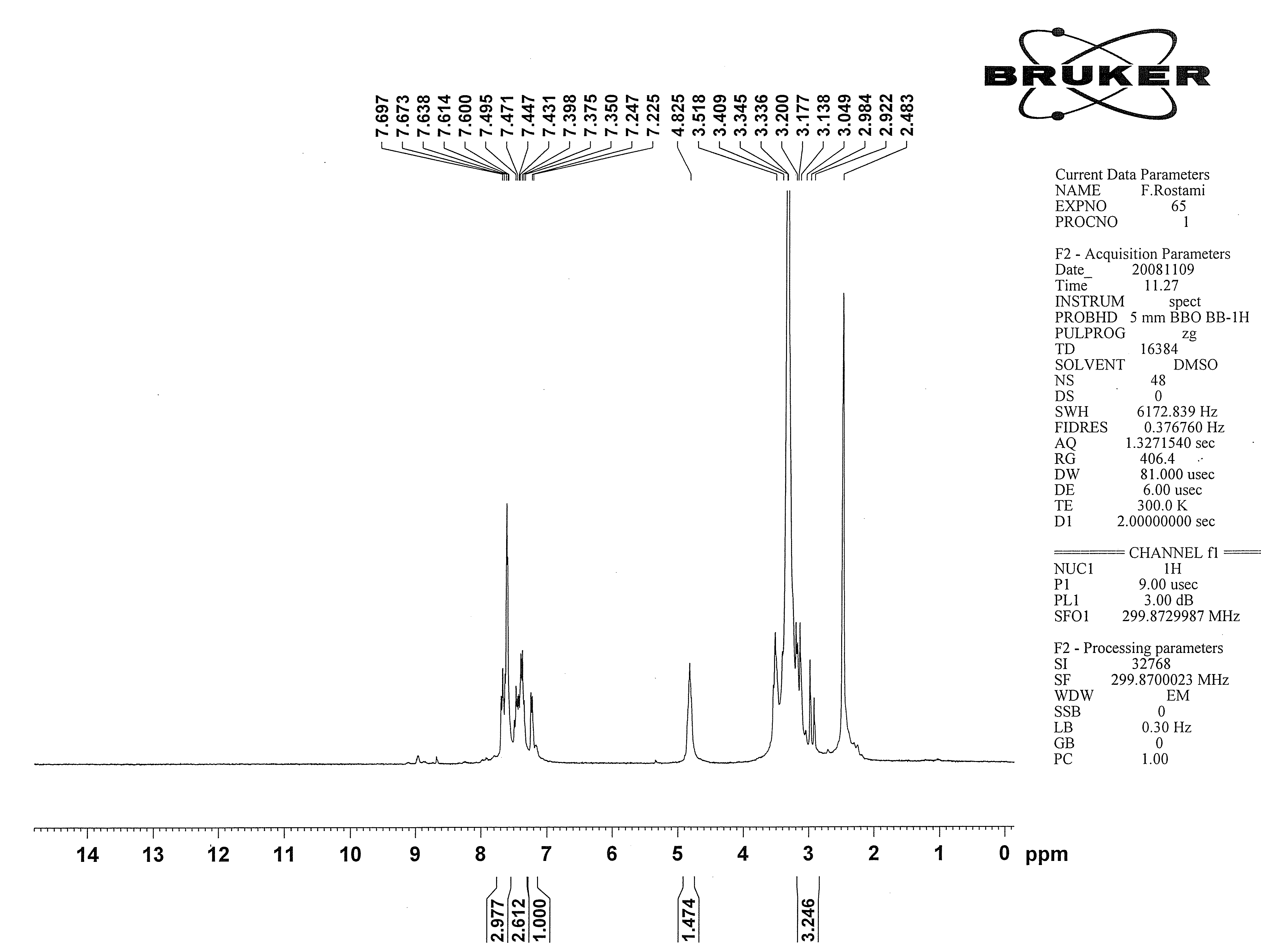
**

**Fig. S4a. ^1^H NMR of 5c**

**
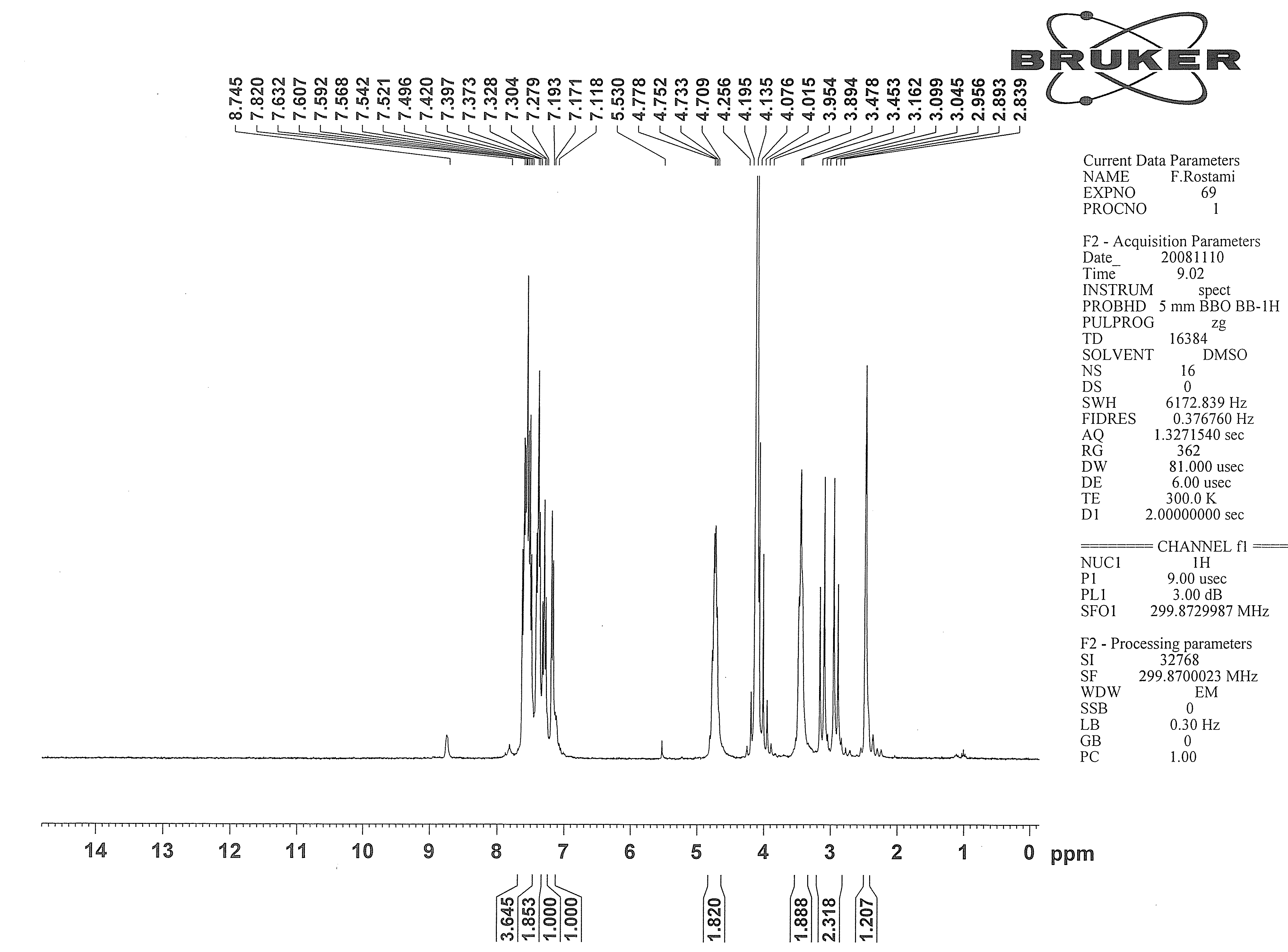
**

**Fig. S4b. D_2_O exchange of 5c**

**
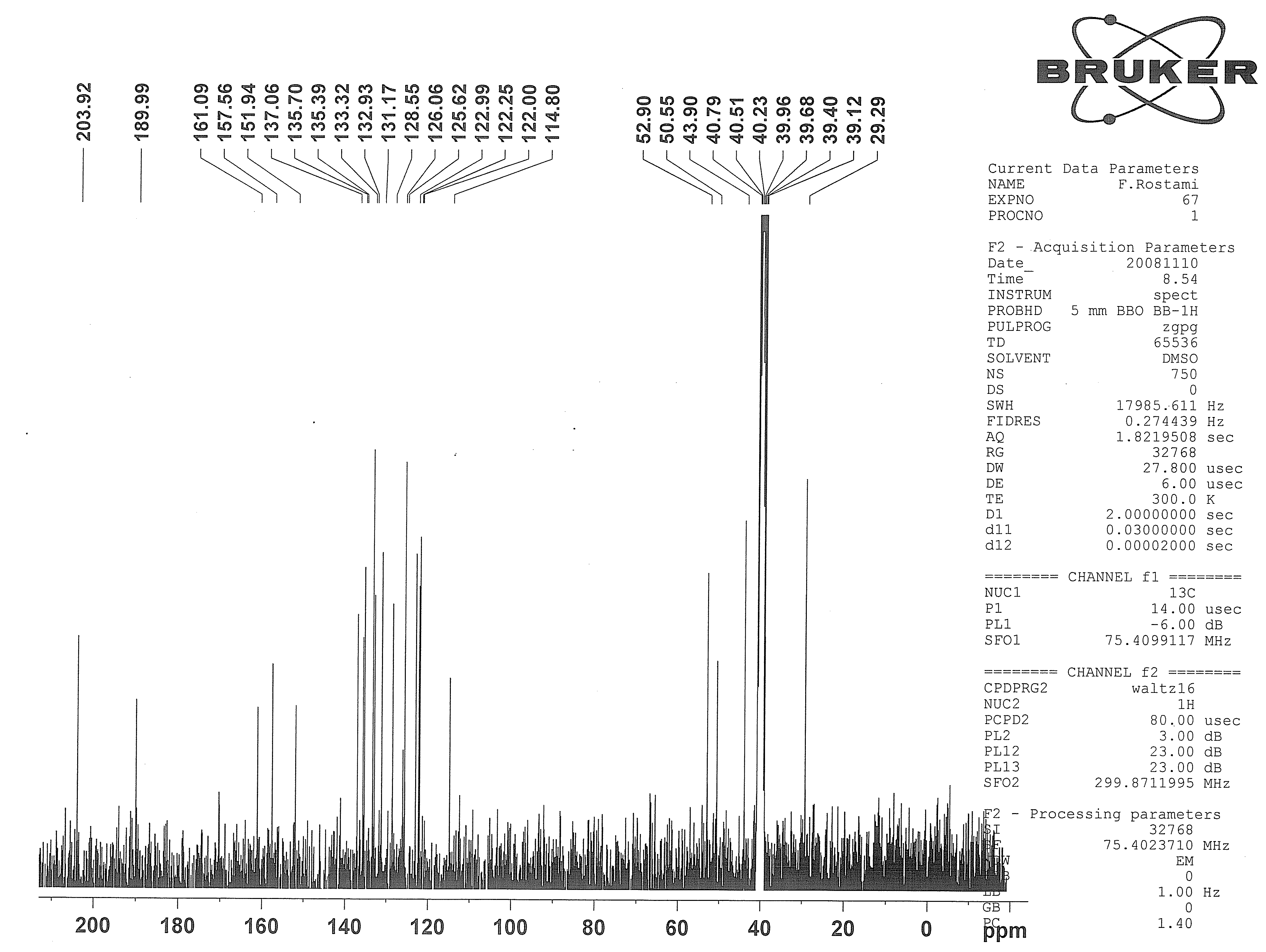
**

**Fig. S4c. ^13^C NMR of 5c**


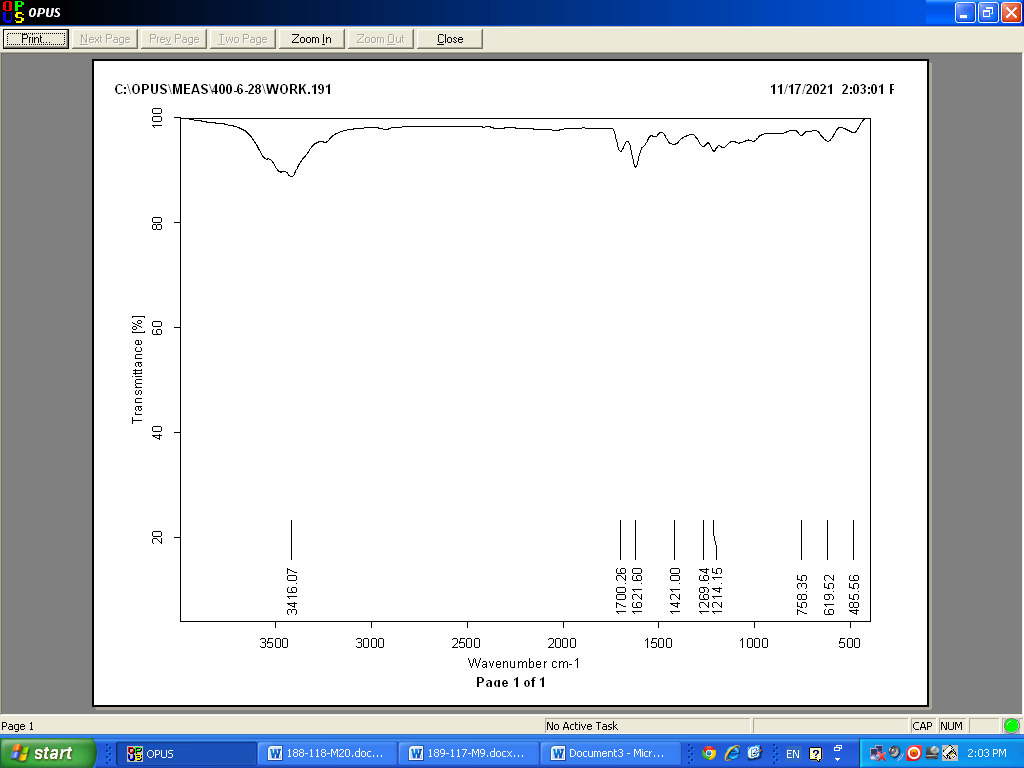


**Fig. S4d. IR of 5c**


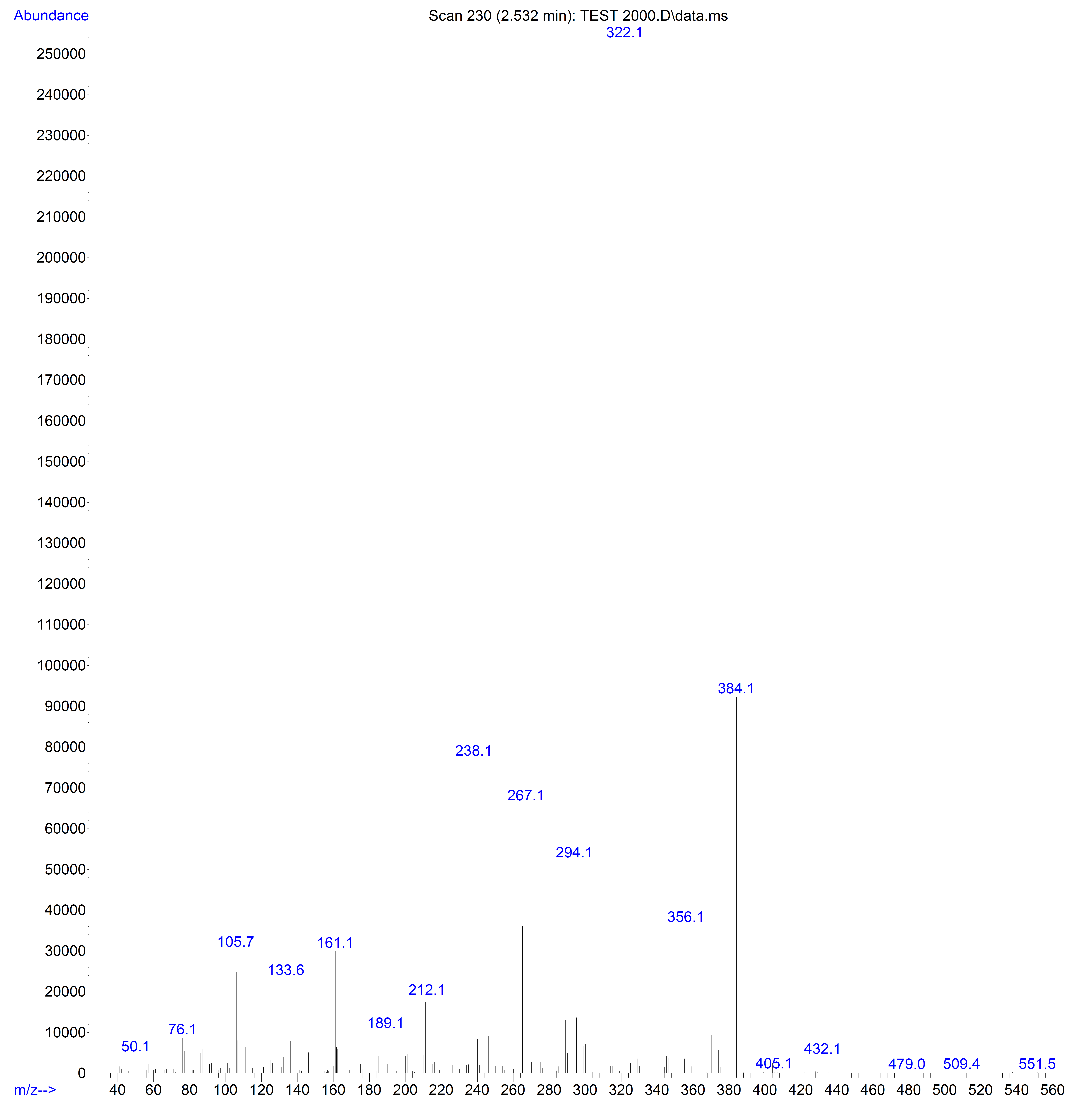


**Fig. S4e. Mass of 5c**

**
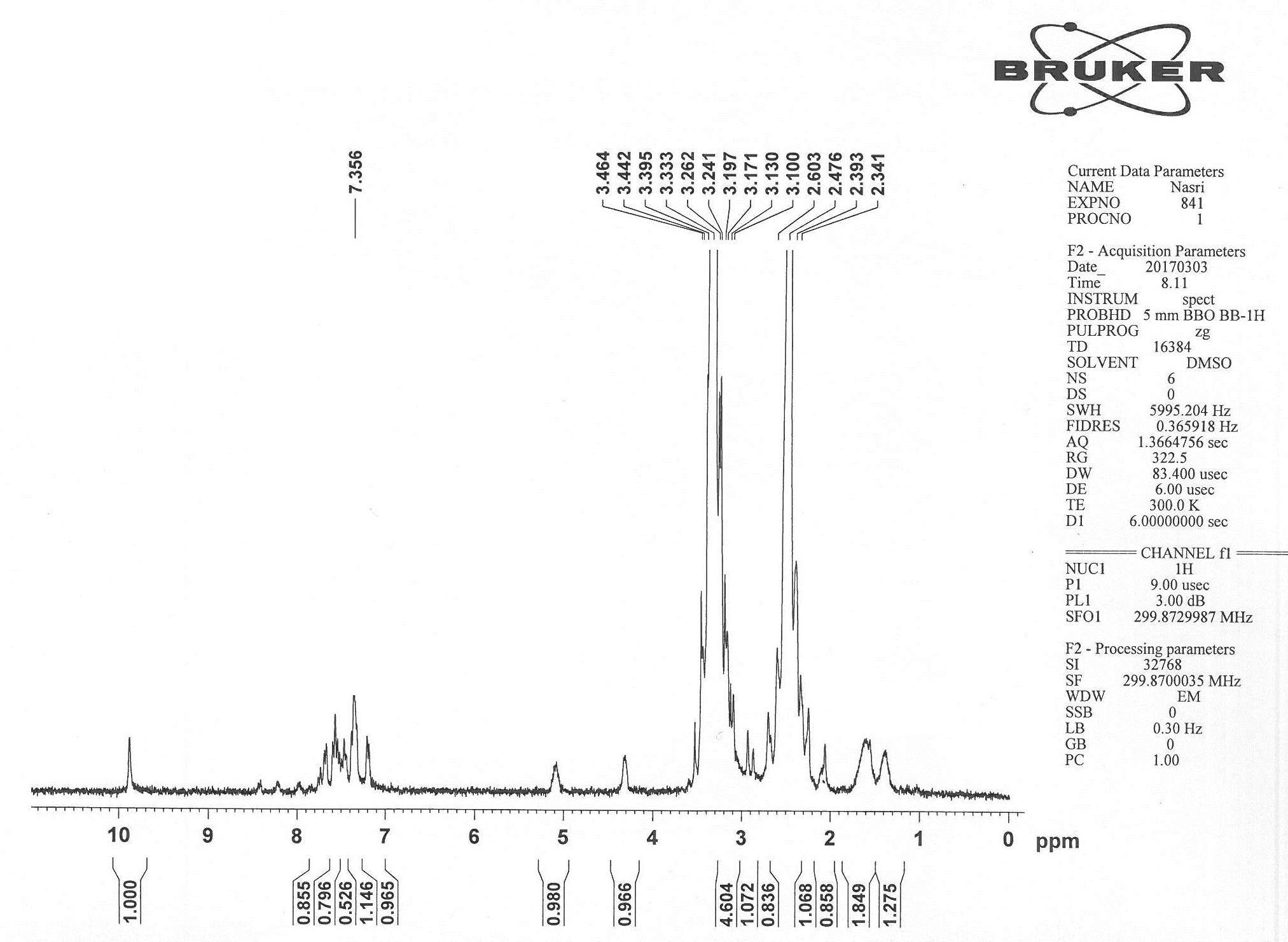
**

**Fig. S5a. ^1^H NMR of 5d**

**
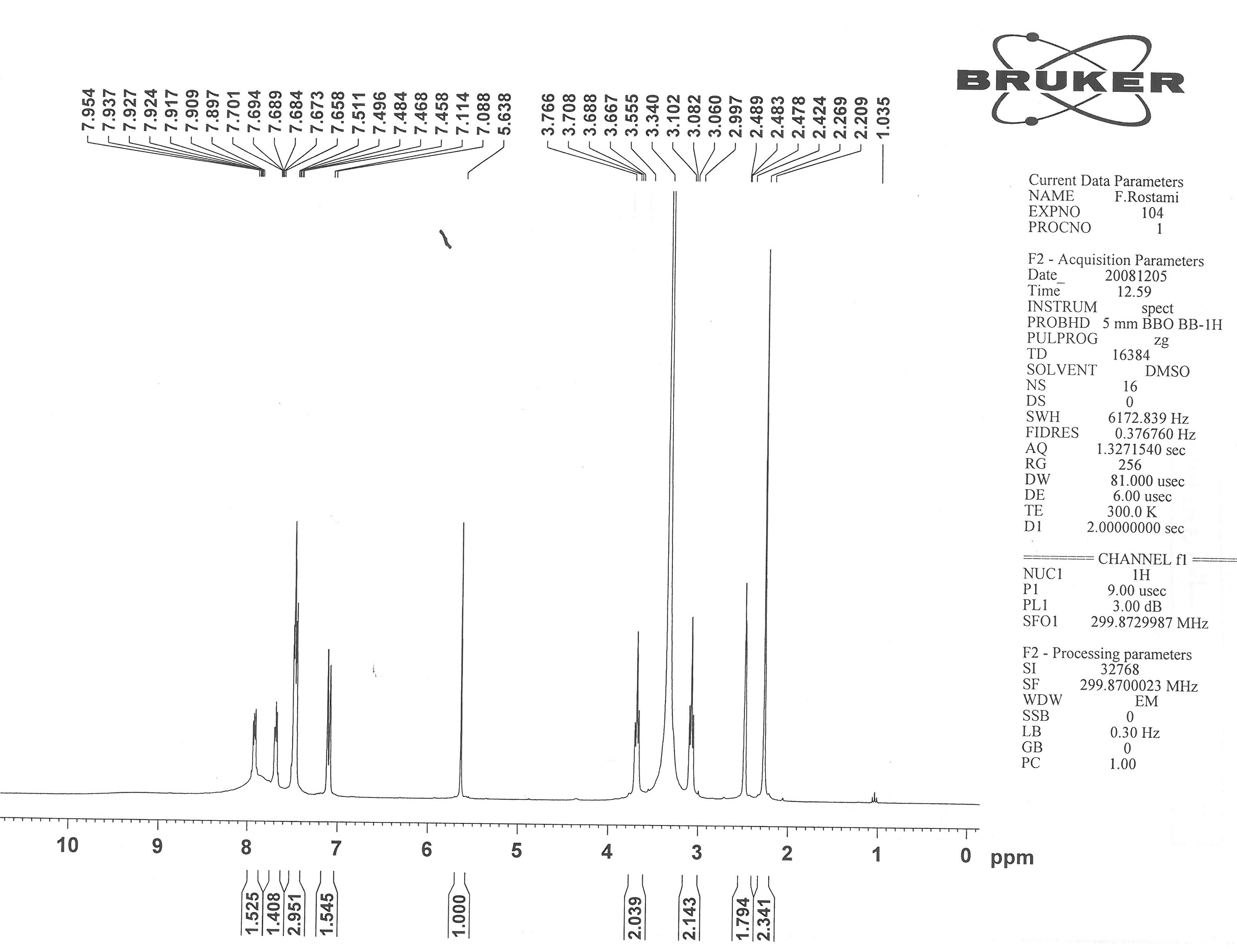
**

**Fig. S6a. ^1^H NMR of 6a**

**
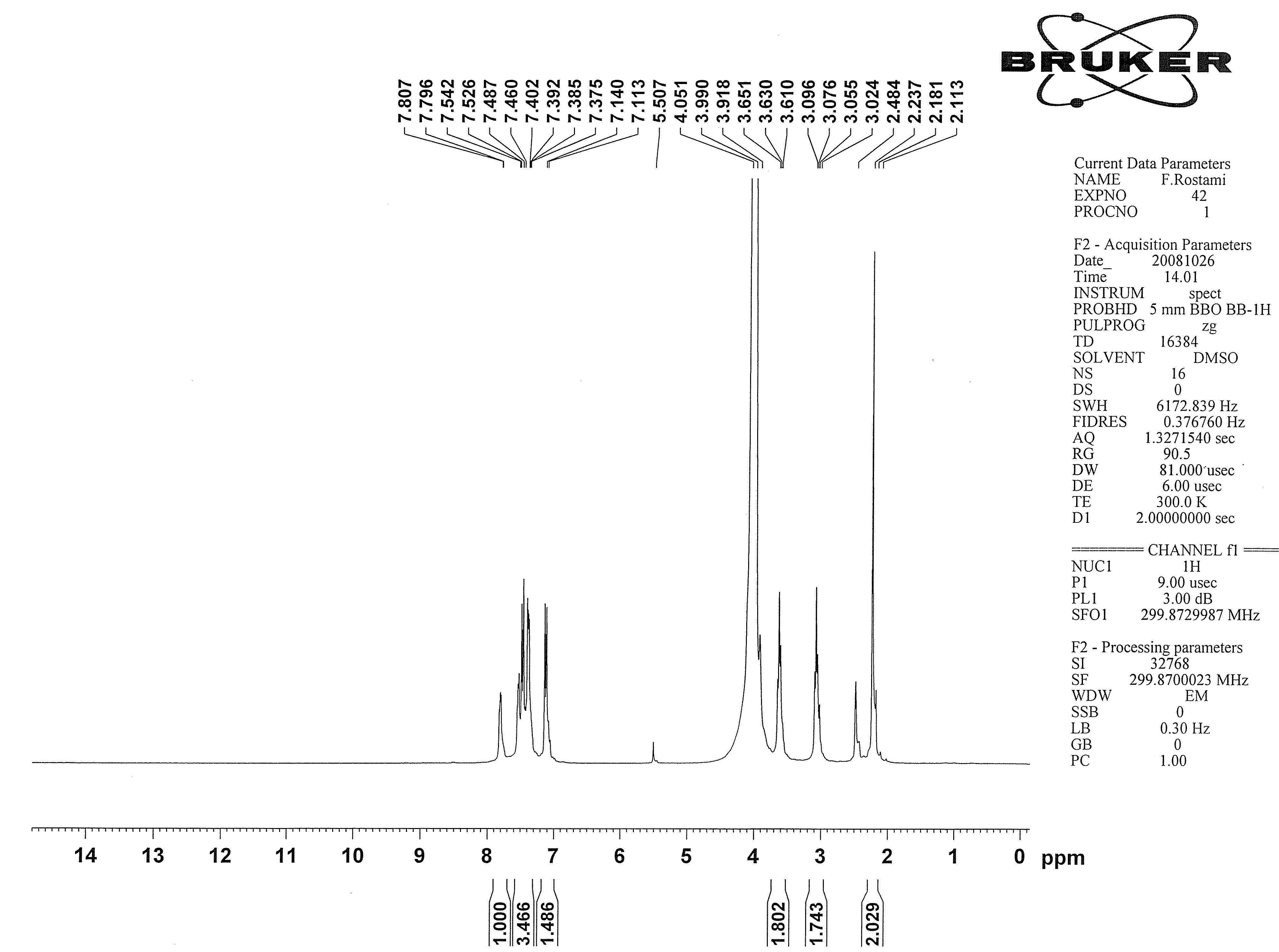
**

**Fig. S6b. D_2_O exchange of 6a**

**
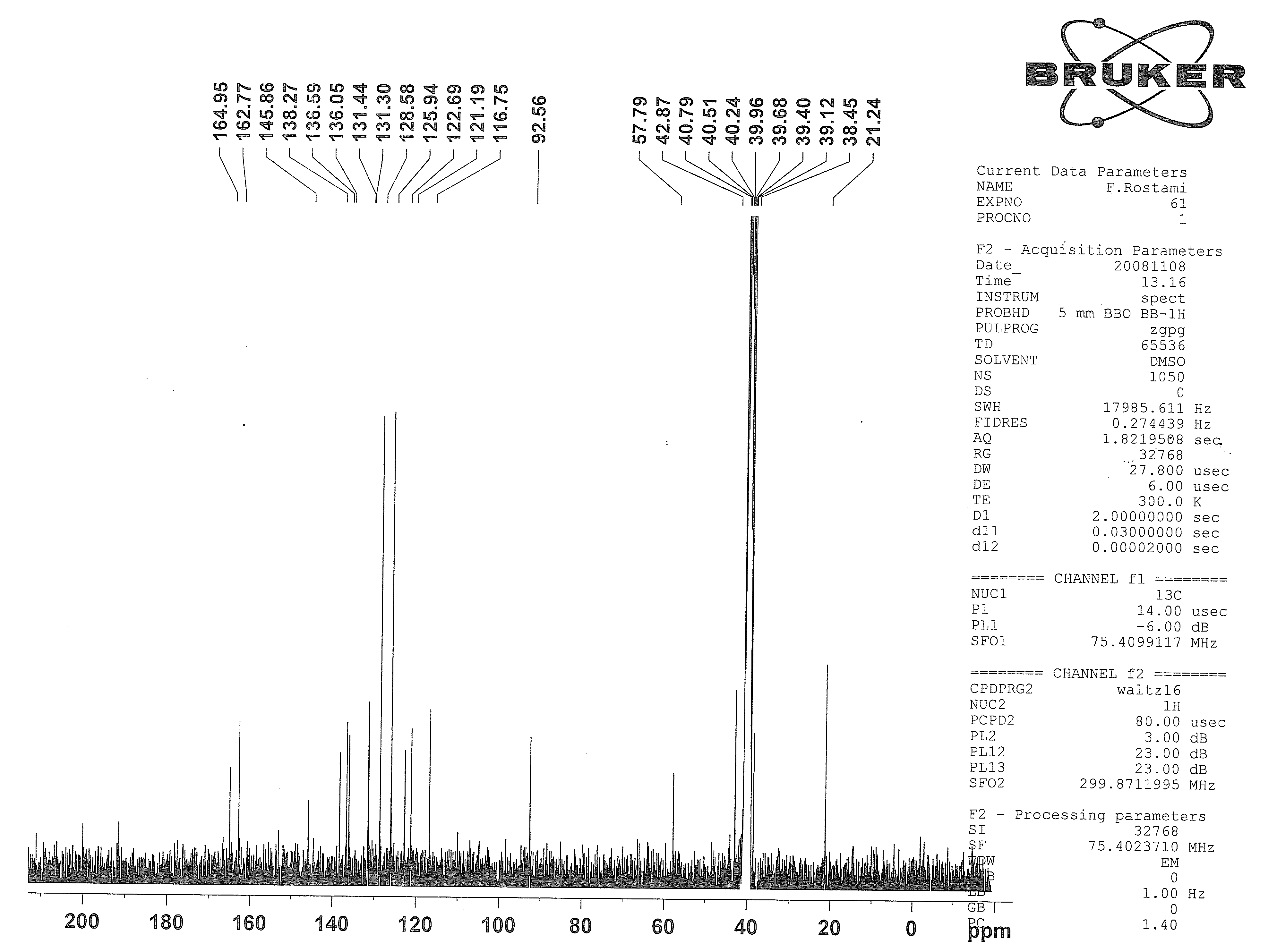
**

**Fig. S6c. ^13^C NMR of 6a**


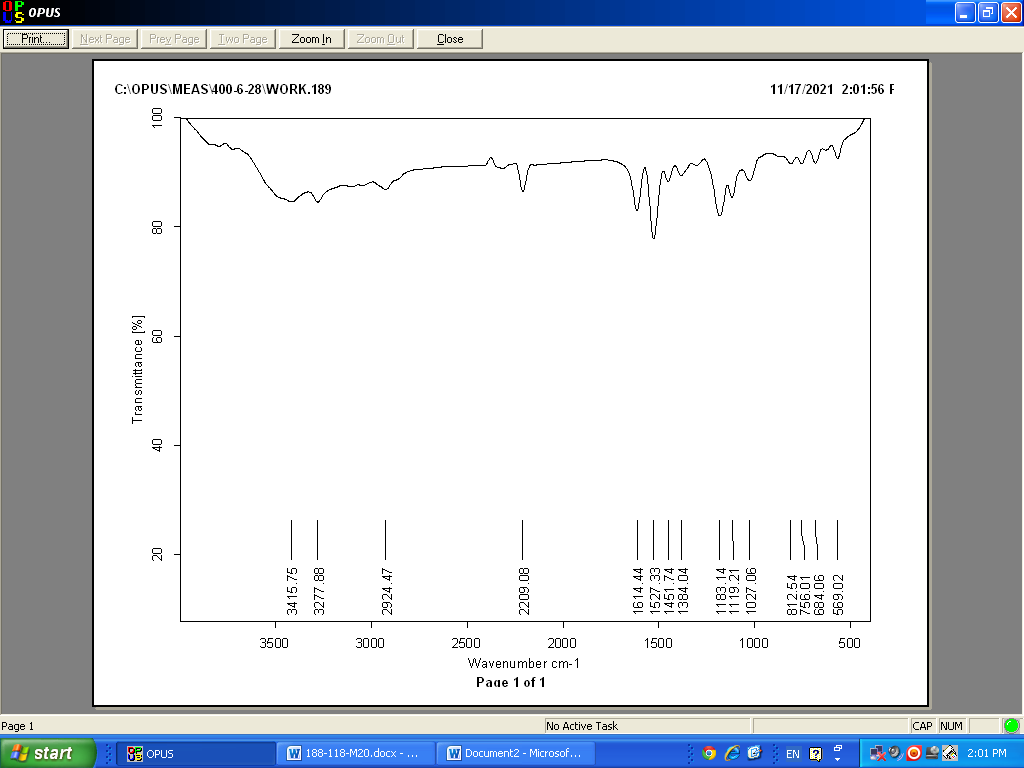


**Fig. S6d. IR of 6a**


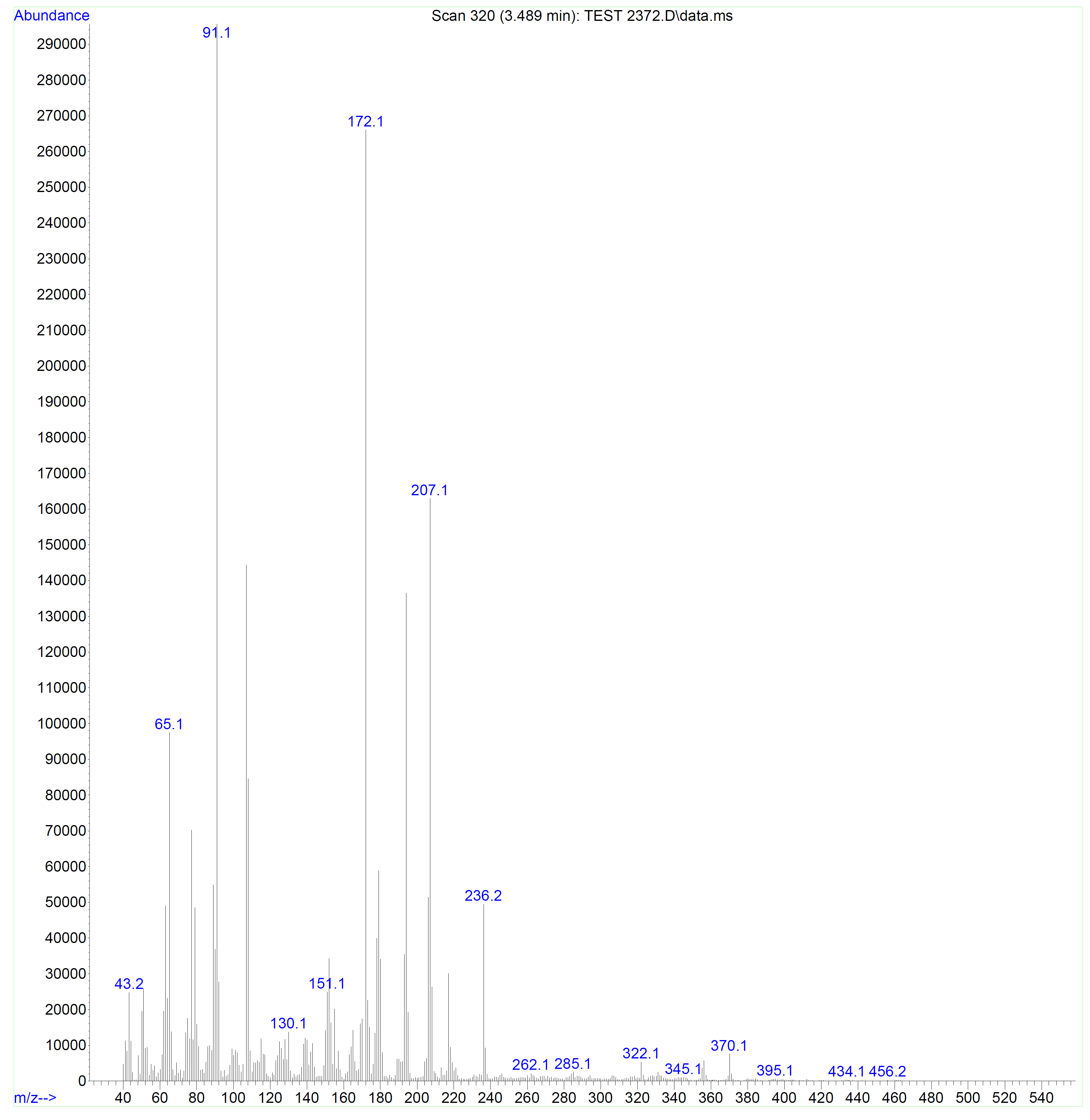


**Fig. S6e. Mass of 6a**


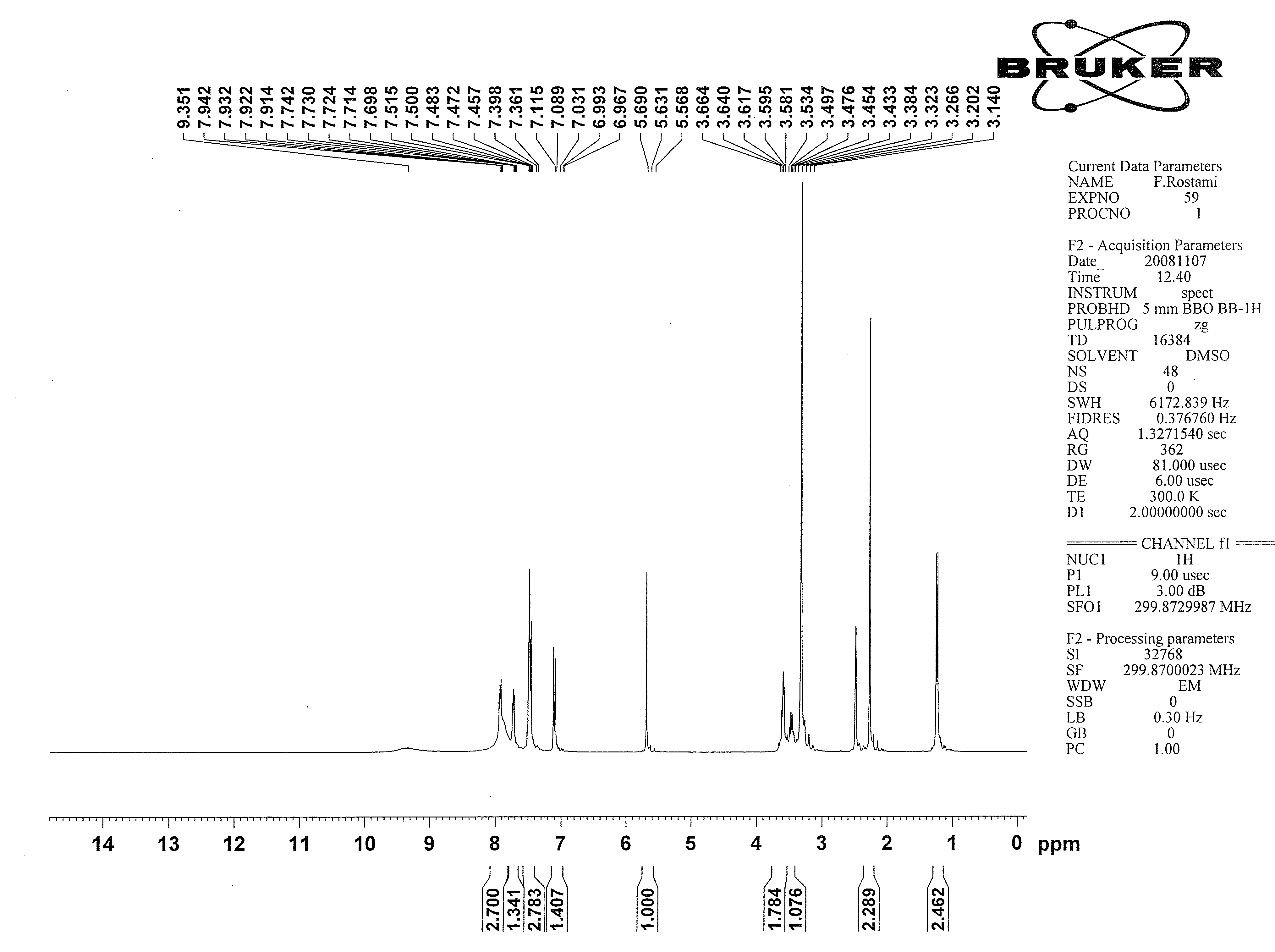


**Fig. S7a. ^1^H NMR of 6b**

**
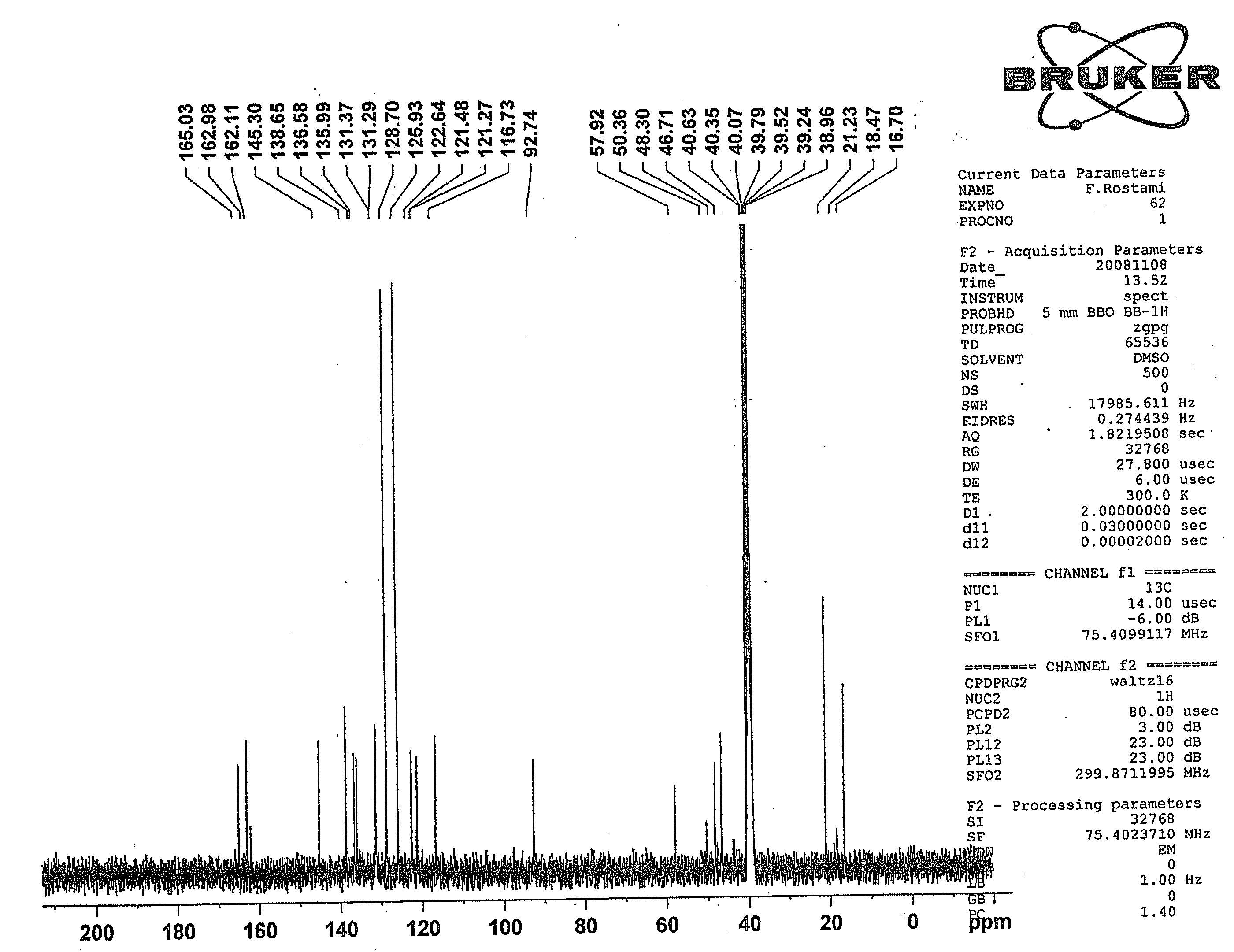
**

**Fig. S7b. ^13^C NMR of 6b**


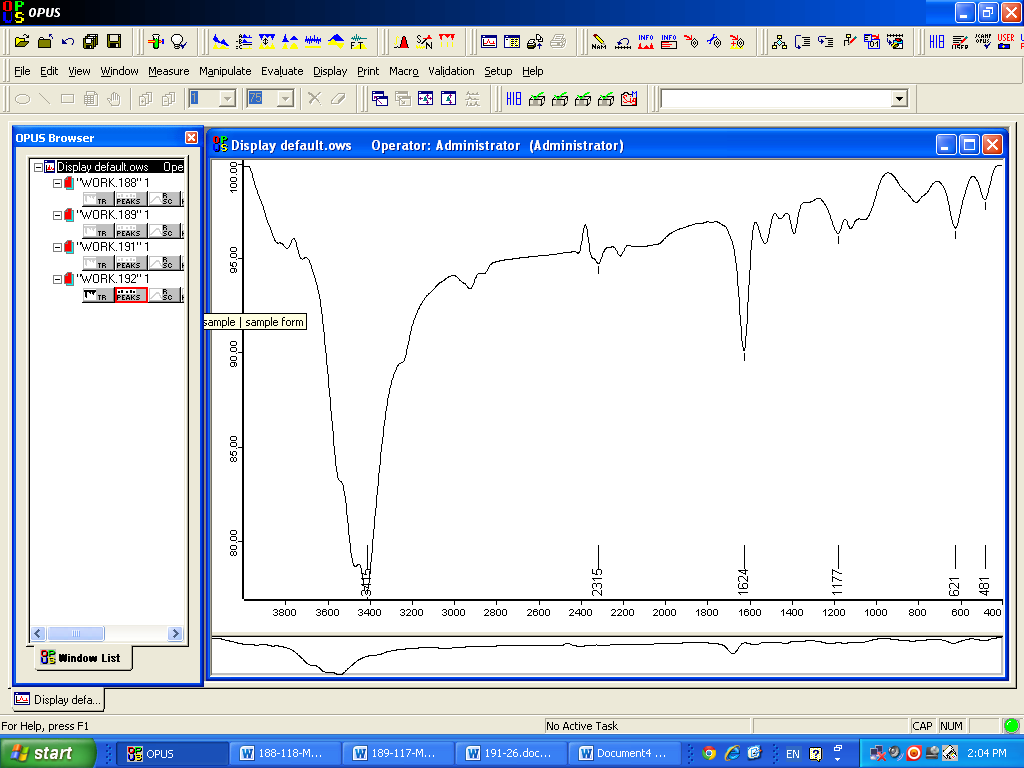


**Fig. S7c. IR of 6b**


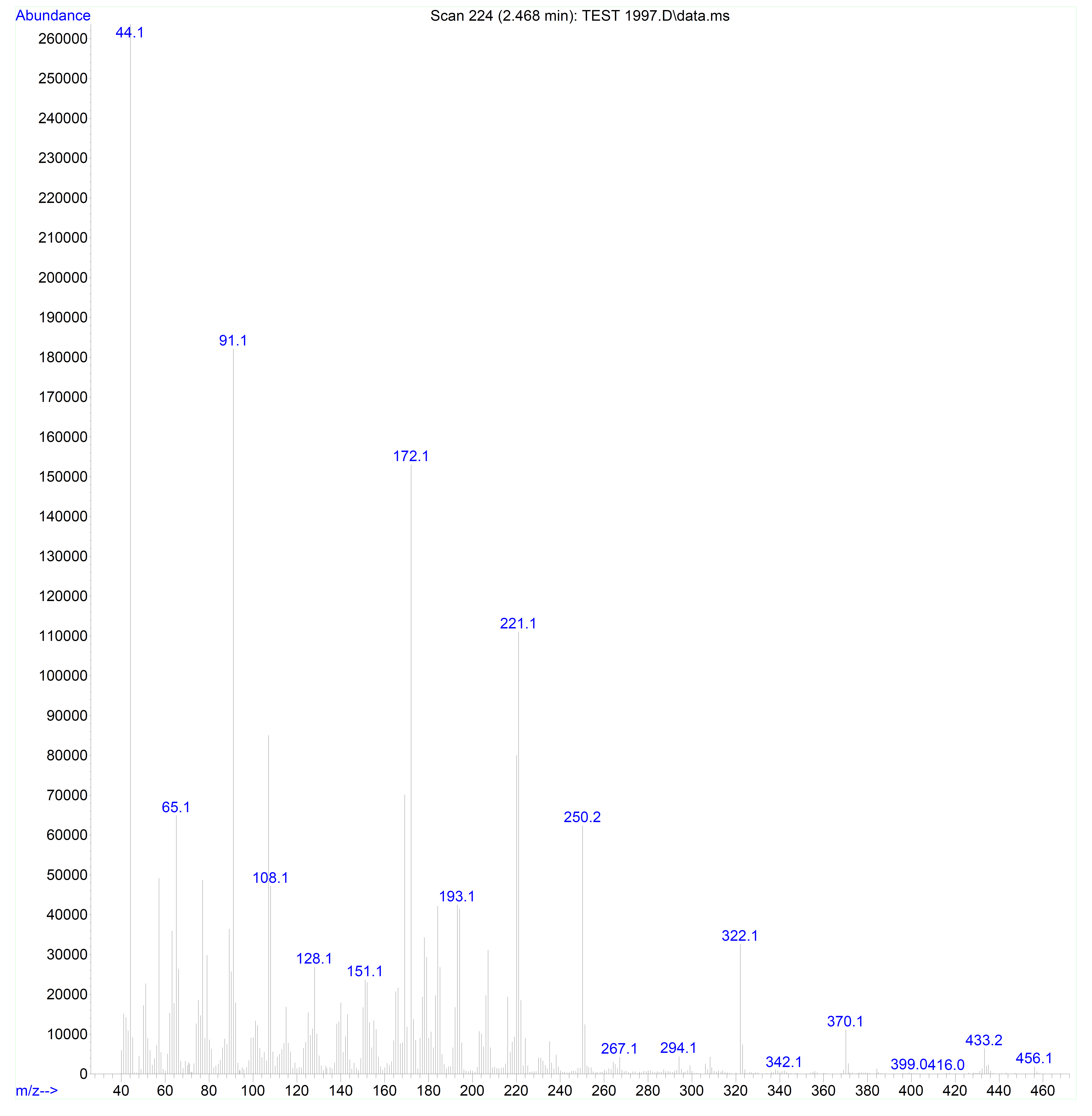


**Fig. S7d. Mass of 6b**


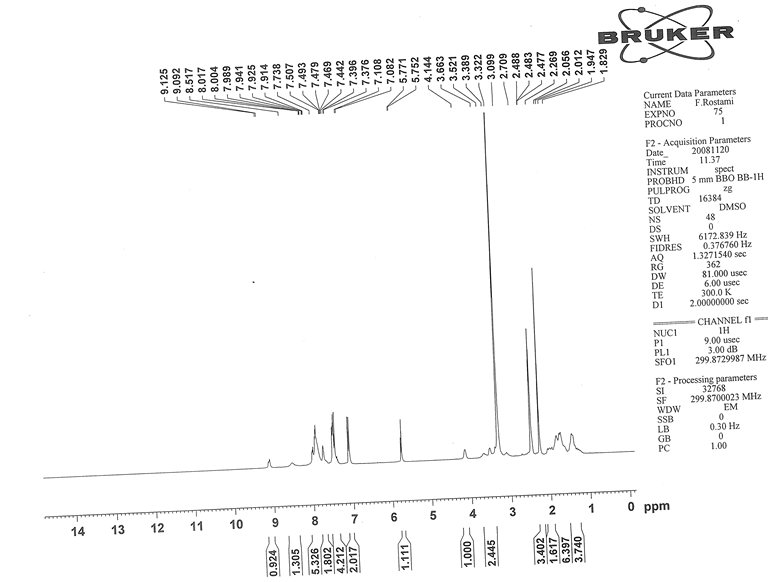


**Fig. S8a. ^1^H NMR of 6c**

**
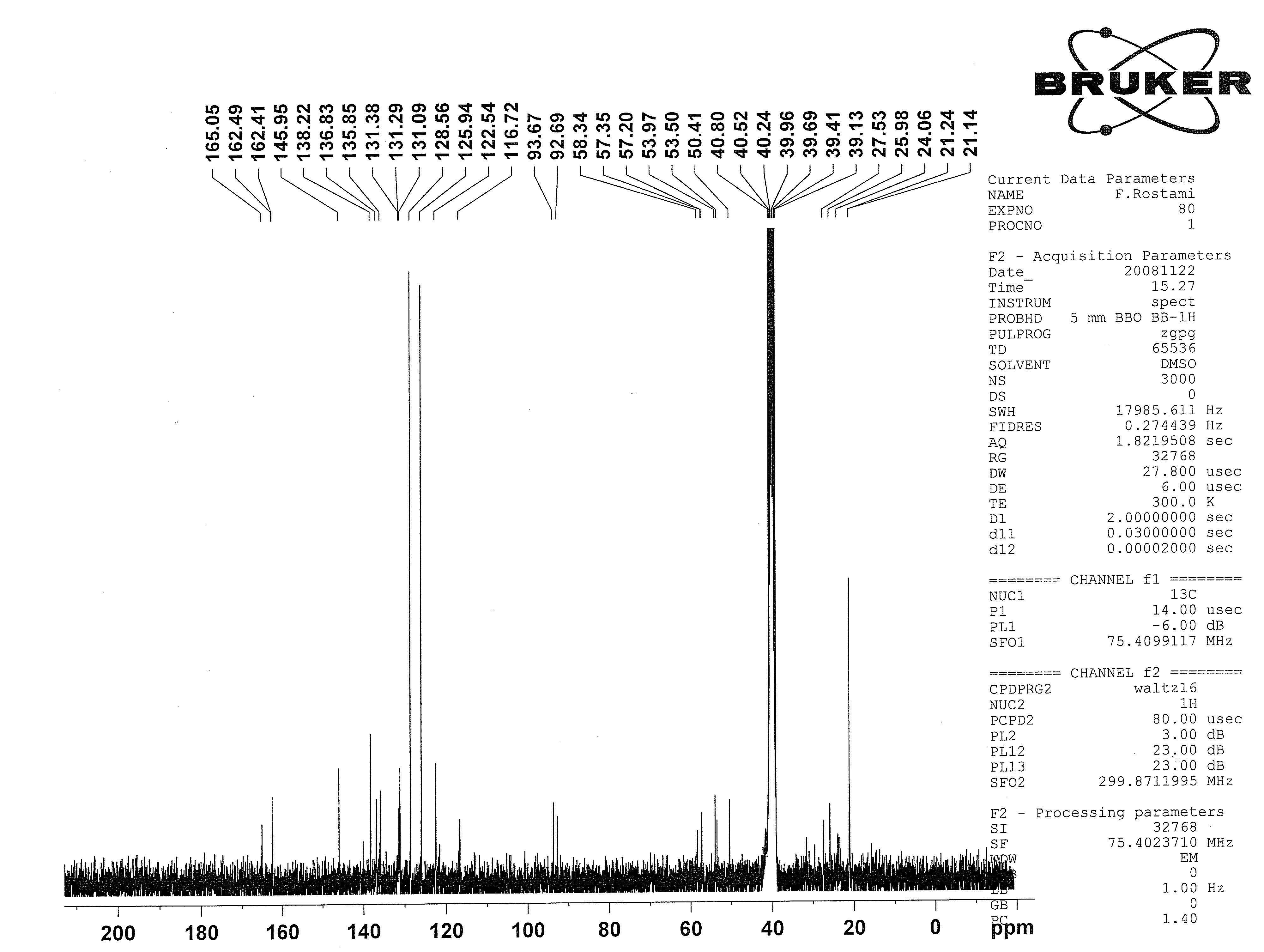
**

**Fig. S8b. ^13^C NMR of 6c**


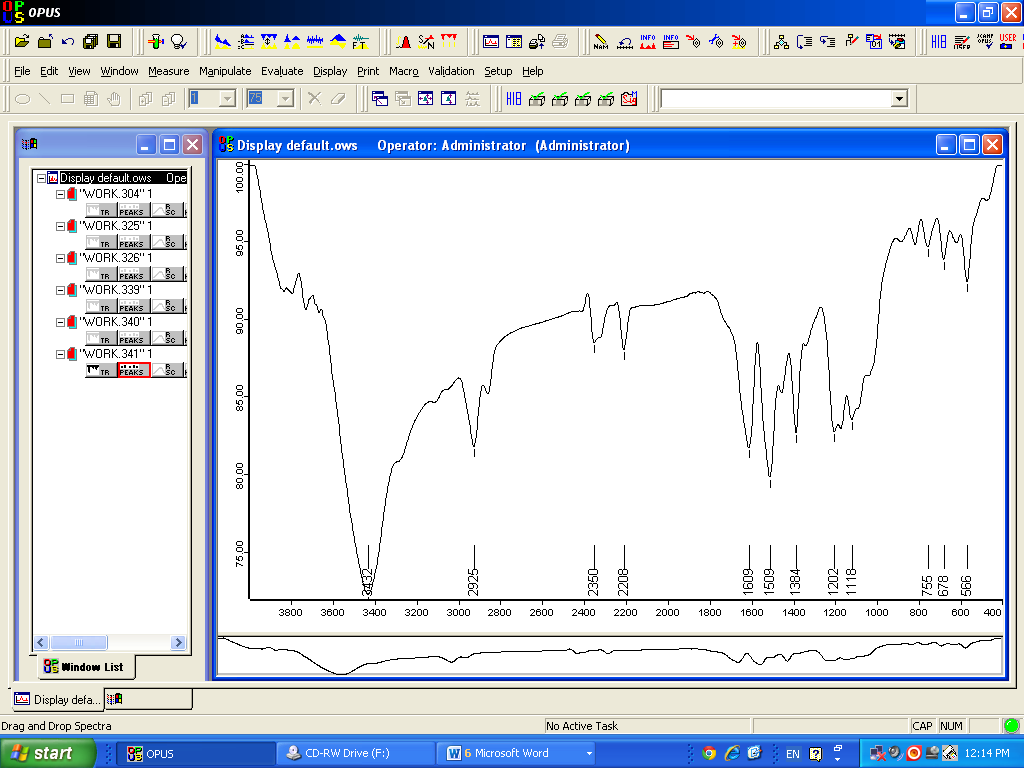


**Fig. S8c. IR of 6c**


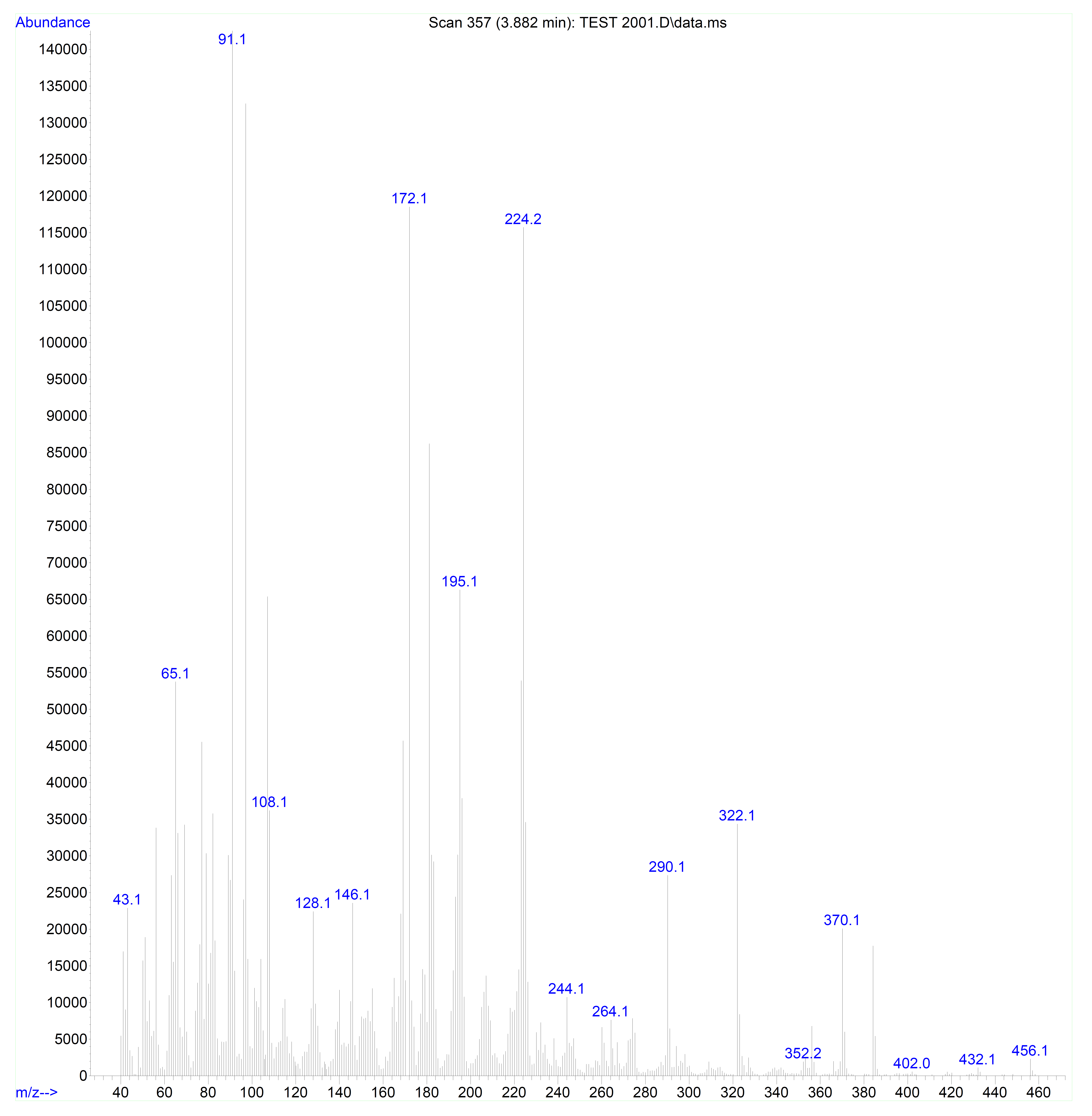


**Fig. S8d.** Mass of 6c


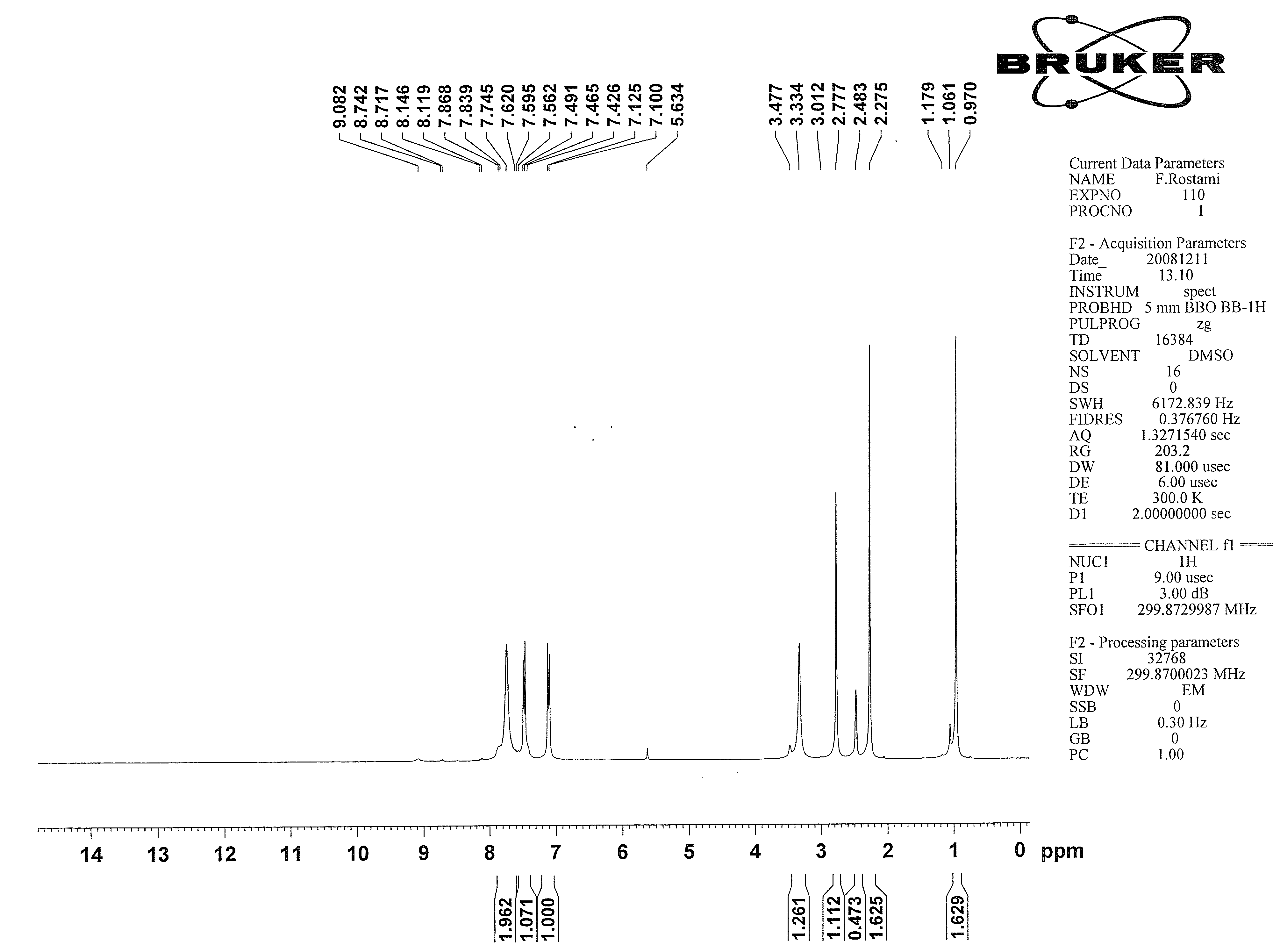


**Fig. S9a. ^1^H NMR of 6d**


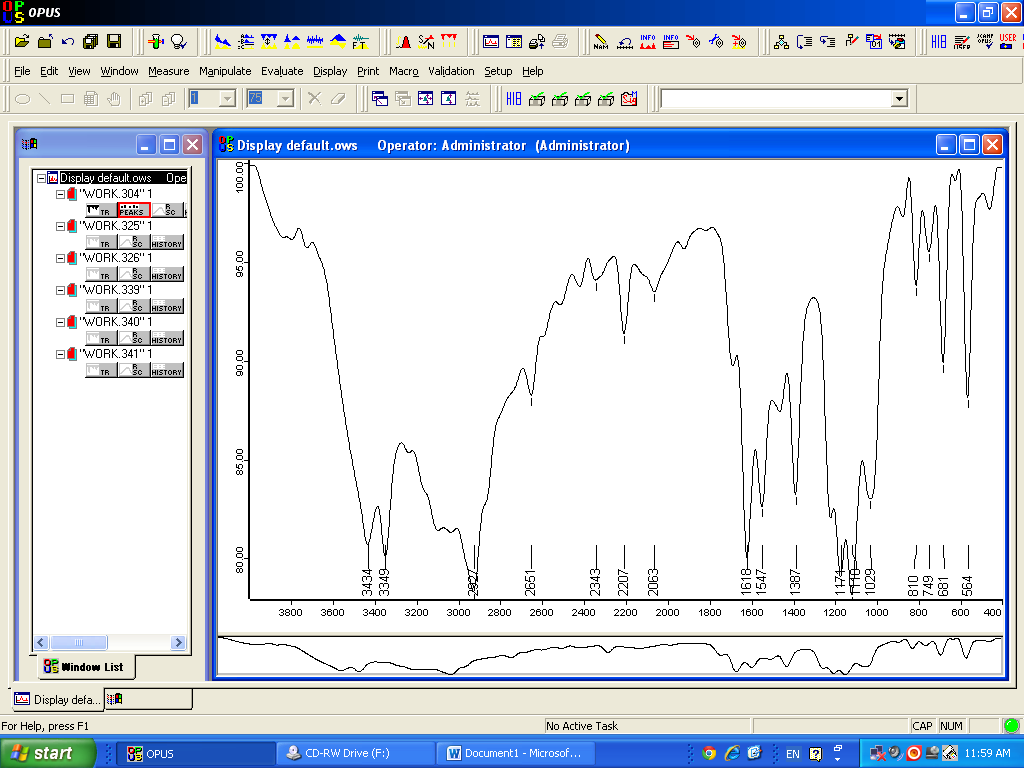


**Fig. S9b. IR of 6d**

**
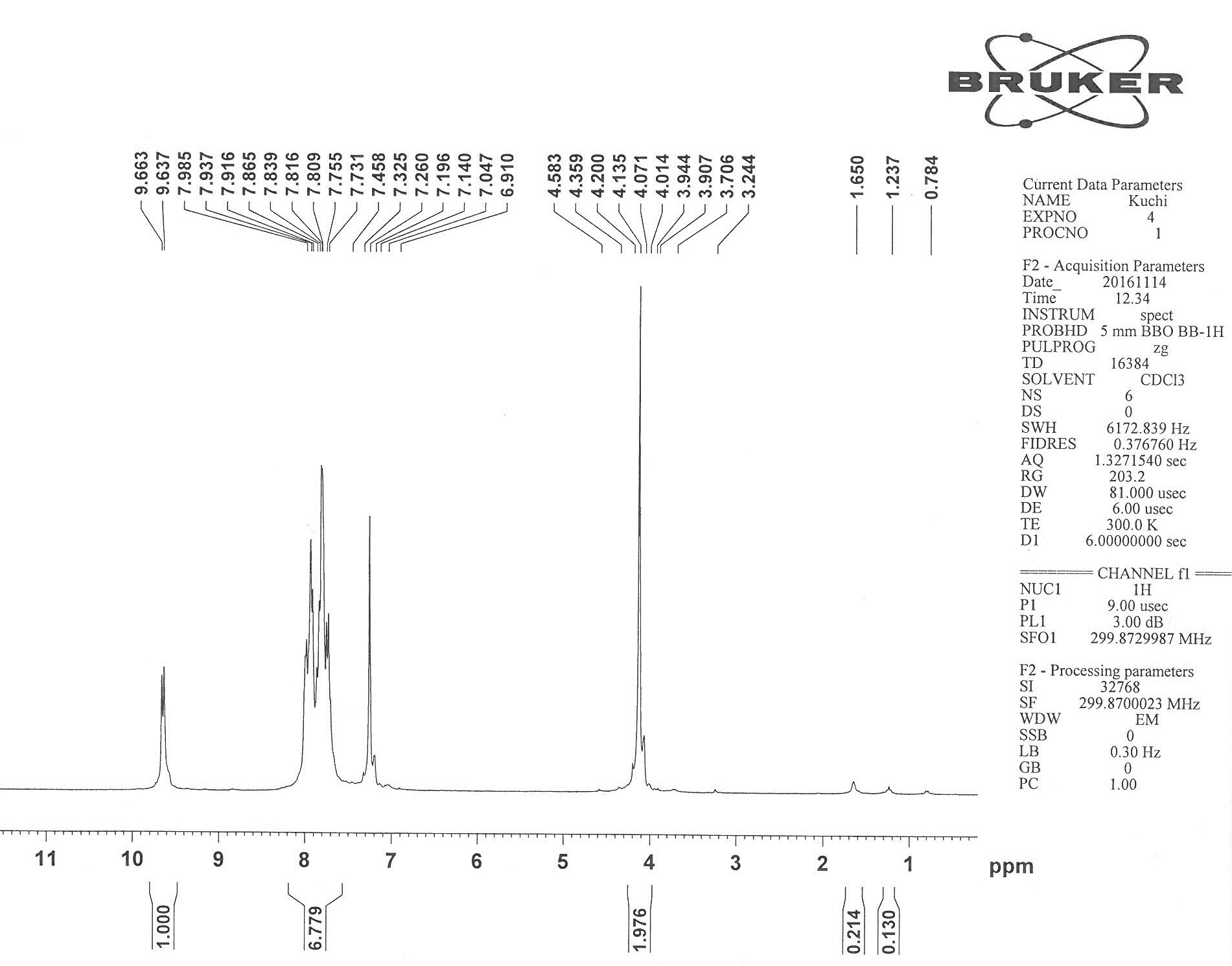
**

**Fig. S10. ^1^H NMR of intermediate II**
